# Prioritizing farm management interventions to improve climate change adaptation and mitigation outcomes—a case study for banana plantations

**DOI:** 10.1007/s13593-022-00809-0

**Published:** 2022-08-04

**Authors:** Eduardo Fernandez, Hoa Do, Eike Luedeling, Thi Thu Giang Luu, Cory Whitney

**Affiliations:** 1 Department of Horticultural Sciences, Institute of Crop Science and Resource Conservation (INRES), University of Bonn, 53121 Bonn, Germany; 2 Escuela de Agronomía, Pontificia Universidad Católica de Valparaíso, Casilla 4-D, Quillota, Chile

**Keywords:** Decision analysis, Embracing uncertainty, Climate-smart agriculture, Farm management interventions, On-farm composting

## Abstract

Intervening into agricultural systems necessarily includes risks, uncertainties, and ultimately unknown outcomes. Decision analysis embraces uncertainty through an interdisciplinary approach that involves relevant stakeholders in evaluating complex decisions. We applied decision analysis approaches to prioritize 21 farm management interventions, which could be considered in certification schemes for banana production. We estimated their contribution to climate change adaptation and mitigation as well as ecological outcomes. We used a general model that estimated the impacts of each intervention on adaptation (benefits minus costs), mitigation (global warming potential), ecological parameters (e.g., biodiversity and water and soil quality), and farming aspects (e.g., yield, implementation costs and production risks). We used expert and documented knowledge and presented uncertainties in the form of 90% confidence intervals to feed the model and forecast the changes in system outcomes caused by each intervention compared to a baseline scenario without the measure. By iterating the model function 10,000 times, we obtained probability distributions for each of the outcomes and farm management interventions. Our results suggest that interventions associated with nutrient management (e.g., composting and nutrient management plan) positively affect climate change adaptation, mitigation, and ecological aspects. Measures with no direct yield benefits (e.g., plastic reduction) correlate negatively with adaptation but have positive impacts on ecology. Creating buffer zones and converting low-productivity farmland (incl. unused land) also have positive ecological and adaptation outcomes. Decision analysis can help in prioritizing farm management interventions, which may vary considerably in their relationship with the expected outcomes. Additional work may be required to elaborate a comprehensive assessment of the underlying aspects modulating the impacts of a given measure on the evaluated outcome. Our analysis provides insights on the most promising interventions for banana plantations and may help practitioners and researchers in focusing further studies.

**Supplementary Information:**

The online version contains supplementary material available at 10.1007/s13593-022-00809-0.

## Introduction

Agricultural systems are complex and diverse. Interventions to these systems usually come with gains or losses, and in many cases the outcomes are highly uncertain. Decision analysis is an interdisciplinary approach that has been developed and adapted to address uncertainty in agricultural development contexts, where many decisions have to be made without scientific evidence (Howard and Abbas 2015; Shepherd et al. 2015). This challenge is particularly pertinent for decision-making in data-poor environment of development contexts (Luedeling and Shepherd 2016). Decision analysis is based on a participatory process where actors with diverse perspectives are invited to discuss and illustrate all the important aspects of a particular intervention (Whitney et al. 2018). The approach allows for a comprehensive system analysis without assumptions of certainty that may oversimplify the complexity of an intervention’s real-world implications. Various forms of information including expert and documented knowledge can be incorporated into a coherent analytical model to make inferences about the anticipated outcomes. Uncertainty is expressed using probability distributions derived from multiple sources of information. These distributions provide not only all plausible values for certain quantities; they also associate these values to the likelihood of occurrence (Howard and Abbas 2015). Given the ability to work with uncertainty, decision analysis is highly suitable for assessing the impacts of complex interventions such as farm management measures oriented to comply with international certification schemes.

Various international certification schemes are available to farms that comply with a set of standards for achieving improvements in both environmental and social dimensions of food production systems. Voluntary Sustainability Standards (VSSs) (including Fair Trade, Global GAP, Organic and Rainforest Alliance) are the most common standards for banana plantations. These standards are motivated by increasing demand for such guidelines in both the producing and importing countries. VSSs play an important role in helping to address the sector’s sustainability challenges. The certified plantations and producers need to comply with the criteria embedded in each standard, which encourages the improvement of agricultural practices (Voora et al. 2020). These criteria include climate change mitigation and adaptation practices such as soil conservation and integrated pest management measures. Standard-compliant banana production demonstrates reduced adverse impacts of climate change and highlights the potential for certification schemes to lower and internalize societal costs (Melo and Wolf 2007; Voora et al. 2020). Improved practices can also play a crucial role in preventing environmental damage (Hernandez and Witter 1996). The market for standard-compliant sustainably grown bananas is growing. However, uncertainty on the cost-effectiveness of complying with certain production standards can be a barrier for resource-constrained producers and may influence adoption decisions.

Bananas are widely grown in tropical and subtropical regions, where they provide dietary diversity and make substantial contributions to the local and national economies of producing countries. While Asia is the largest banana-producing region, Latin America and the Caribbean are the largest exporting regions. More than 90% of the bananas produced in Latin America and the Caribbean are exported (Varma and Bebber 2019), constituting approximately 80% of all global banana exports (Voora et al. 2020). While climate change impacts threaten most agricultural systems, planetary warming may favor some banana production areas. Increasing temperature has been projected to increase yield in some regions (Varma and Bebber 2019) and expand feasible areas for cultivation by 50% by 2070. Many banana growing regions, on the other hand, are prone to climatic hazard events such as extreme rain, strong wind, and severe drought. Pest and disease incidence is also a common threat in banana plantations. Biotic stress problems are aggravated by the prevalence of monocultures composed of clones of the commercial “Cavendish” cultivar, which accounts for about 76% of bananas grown throughout Latin America (Varma and Bebber 2019). Monoculture production models with intensive chemical input significantly degrade the environment. Air, soil, and water quality may be adversely affected by the frequent application of chemical inputs, especially pesticides (Hernandez and Witter 1996; Henriques et al. 1997; Castillo et al. 2000), as well as by other cultivation and post-harvest management practices (Russo and Hernández 1995; Hernandez and Witter 1996; Bellamy 2013; Padam et al. 2014). Banana producers are therefore faced with local and international pressure to implement environmentally friendly and less chemical-intensive production practices (Bellamy 2013).

Here, we provide a systematic approach to assessing the likely effects of a number of farm management measures, which could be considered in certification schemes, on climate change adaptation and mitigation as well as environmental impacts at farm-level. We used complex banana production systems as a case study to prioritize 21 interventions regarding their expected effect on yield, production costs and risks, global warming potential, and environmental outcomes. We demonstrate a novel approach for supporting certification schemes aiming to advance climate change adaptation and mitigation. Working with available expert knowledge and other data sources, we generate simulations and make forecasts that allow us to offer recommendations for certification schemes that help address current and future climate- and environment-related challenges. We offer advice for producers, certifiers, importers, and development aid organizations. We demonstrate the application of holistic approaches that are effective for working with complex systems, incorporating uncertainty and systems understanding. The approaches are effective for generating quick, actionable decision support for difficult and risky certification schemes.

## Materials and methods

### Framework description and initial system understanding

**Fig. 1:**
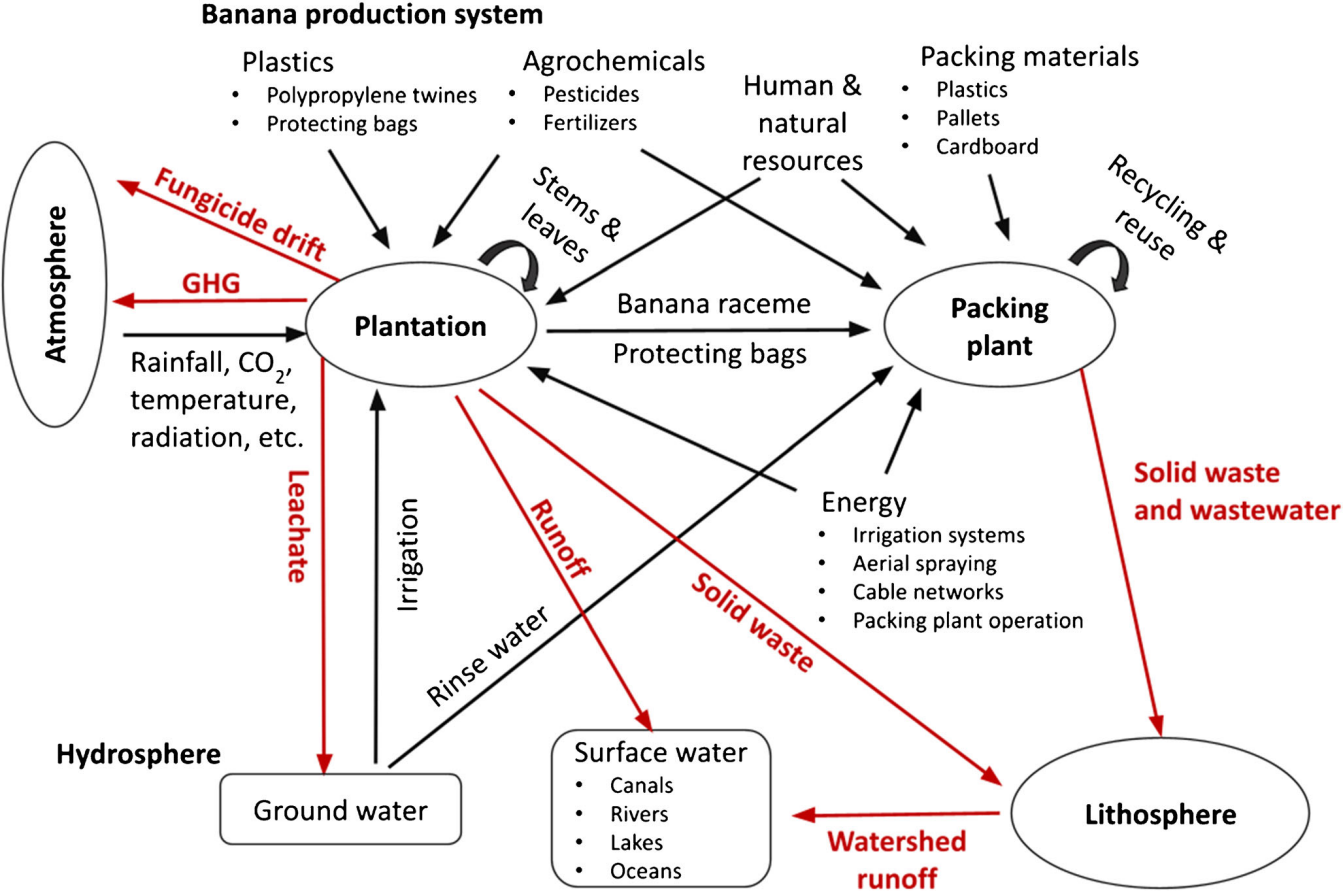
Framework describing a typical export-oriented banana plantation in Latin America, including internal and external components of the production system. The figure characterizes components of the production system and interactions with the system’s surrounding environment (i.e., atmosphere, lithosphere, and hydrosphere). Incoming arrows indicate the inputs to the components, outgoing arrows represent outputs from the components. Red arrows are the main environmental problems that the proposed farm management interventions are expected to address. We adapted this figure from Hernandez and Witter (1996).

Banana production systems are typically composed of two main production units: the banana plantation and the packing plant (Fig. 1). Both units require natural resources such as soil, water, temperature, and sunlight and management inputs such as labor, agrochemicals, energy, and plastics. All these resources produce not only consumable products but also undesired by-products including solid waste, chemical residue, and greenhouse gases (GHGs). Without adequate treatment, these products can contaminate the surrounding ecosystems through surface runoff, leaching, and air drift (Hernandez and Witter 1996).

To assess interventions aiming to address major concerns of banana production systems, we based our study on the work of the Action Alliance for Sustainable Bananas (ABNB) of the German Corporation for International Cooperation (Deutsche Gesellschaft für Internationale Zusammenarbeit; GIZ). ABNB is a dialog and action forum that gathers representatives of multiple stakeholder groups to discuss and develop sustainable banana supply chains. As part of its actions, ABNB seeks to identify interventions to address climate change adaptation, mitigation, and environmental concerns of banana production. They seek measures that are currently available to most farmers, are effective, are cost-effective, and pose low risks. The ABNB team shared an initial list of options from different certification systems such as Rainforest Alliance, Fair Trade, Global GAP, and Organic. We summarized the original list of options by identifying common goals and requirements among certification schemes and derived five main groups of actionable measures (21 specific interventions; see Annex 1 in supplementary materials). The interventions and recommendations described here are focused on large-scale export-oriented banana production systems in the humid tropical climates of Latin American countries. Banana plantations in Latin America are usually monocultures composed of clones of the commercial “Cavendish” cultivar (Varma and Bebber 2019) and cultivated in patches of deep rich soil in lowland floodplains (Hernandez and Witter 1996). These monocultures often suffer from high pressure of pathogens (e.g., bacteria and fungi) and insects, which reduce the yield potential of the crop (Hernandez and Witter 1996).

**Fig. 2:**
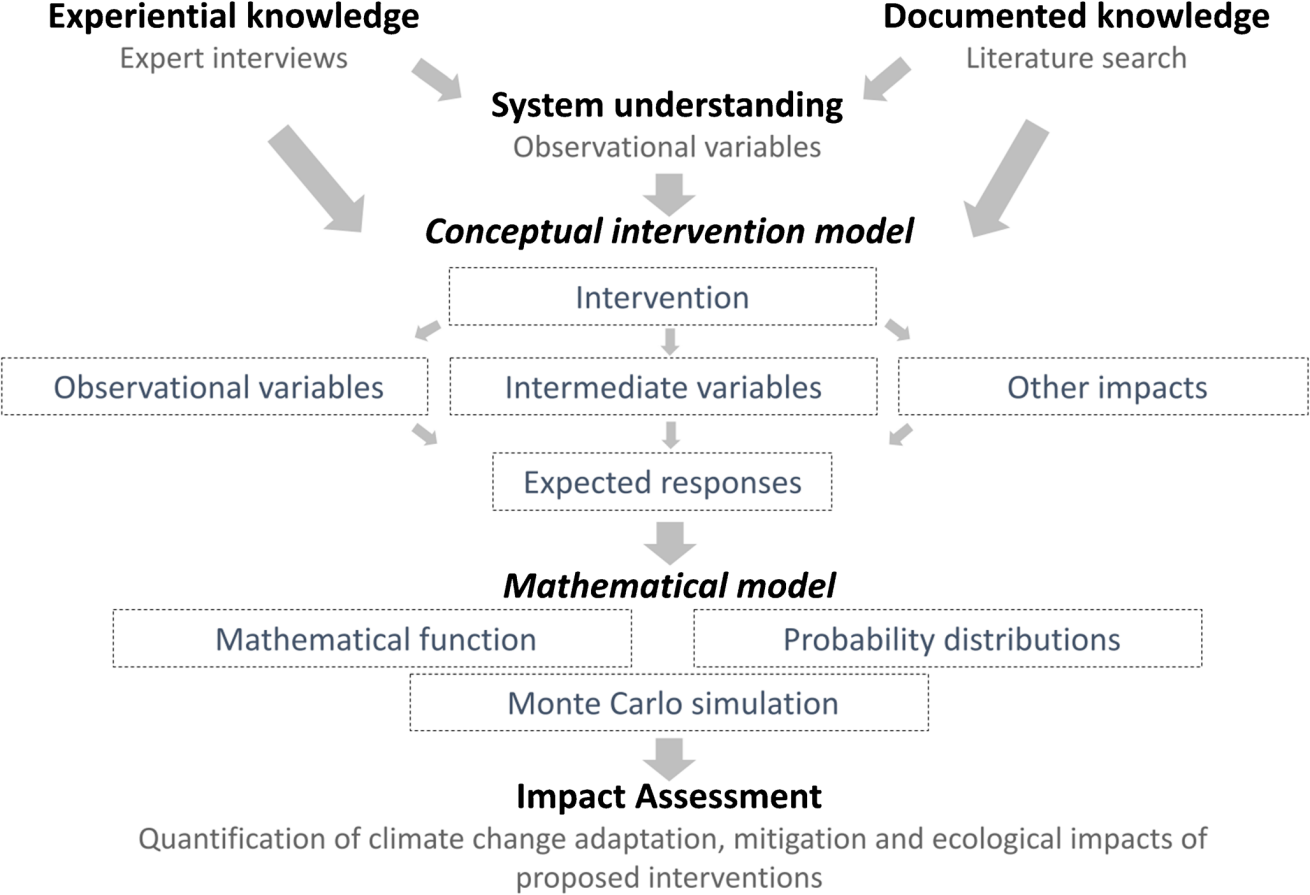
Schematic framework for an impact assessment of farm management interventions for climate change mitigation and adaptation as well as ecologically sustainable production of commercial banana plantations in Latin America.

We applied the principles of decision analysis to produce a general impact assessment for the interventions aiming to support sustainable export-oriented banana production (Fig. 2). We used all available sources of information to understand the targeted system and the possible implications of the various farm management interventions. This included an extensive literature review on banana production and climate change adaptation and mitigation. We created diagrams defining the production systems and the expected impacts of certification requirements and recommendations. We frequently updated these diagrams throughout the project, as we gathered greater understanding of the system.

After defining the general framework for the impact of the farm management interventions, we set out to capture the expertise and knowledge of experts in the fields of certification, banana production, and climate change adaptation and mitigation. Given the traveling restrictions due to the coronavirus pandemic, we did not implement a workshop with stakeholders to jointly develop the impact pathways but involved them in the process individually. Their role, therefore, was to provide us with their expertise and knowledge to improve our prior understanding of the system. We used a questionnaire (see Annex 3 in supplementary materials) for expert interviews with 18 participants to better describe the impact mechanisms of measures, in cases where our information was incomplete or missing (e.g., how likely it is that crop residues at the packing plant are composted and by which method).

We formulated the questionnaire for two main aims: (1) to obtain information (e.g., relevant variables) from experts to update our general understanding gained from literature review and preliminary research, and (2) to clarify specific relationships between interventions and intended outcomes (e.g., how effective is composting in practice in large-scale banana production systems). In this questionnaire, we asked the experts about the impacts of the measures on the production system. We also asked them about common risks for banana plantations, which were mostly associated with extreme climate conditions. We customized our questionnaire to match the expertise of each respondent. Selected experts included professionals, researchers, and practitioners working in banana production, certification, food safety and compliance, sustainable agriculture, and import and export. The experts were selected through expert network channels shared with us by ABNB. In general, experts were keen to take part in the questionnaire and they were positive about the use of decision analysis approaches to prioritize farm management interventions in banana plantations. It should be noted, however, that we did not formally analyze the results of the questionnaire but rather used all raw information that we considered helpful in improving our conceptual framework.

Using what we derived from the literature and the feedback from experts, we constructed conceptual frameworks illustrating the impacts of measures in terms of climate change adaptation and mitigation and ecological effects (Figure S1 and S2). The resulting impact pathways served as guidance for constructing a mathematical function estimating the relative effects of each measure on climate change adaptation and mitigation as well as the environment (available at https://github.com/CWWhitney/Certification_Prioritization).

### Mathematical implementation of the graphical impact pathways

#### General model function

We elaborated a general model function that was later applied to each of the proposed farm management interventions to obtain customized mathematical models. Such a customization was achieved by feeding the general function with specific model inputs according to each intervention. For model inputs, we estimated the relative differences in terms of costs, benefits, and other implications of measures in relation to the production systems without those actions (i.e., costs of production with composting vs costs without). We used inputs such as the relative increase in production cost, relative benefit (if any) in yield, and additional benefits associated with the measure (e.g., reduction in energy use). Additionally, we accounted for typical production risks and damage reduction due to measure implementation as well as negative effects or risk increases and yield reductions associated with the measure. Regarding mitigation factors, we defined a parameter to introduce directly any relative effect of the intervention on global warming potential. Similarly, regarding the impacts of a given measure on ecological aspects, we considered the relative impacts on soil and water quality, and biodiversity richness. Finally, we obtained seven metrics as outputs from the function, including (1) relative change in yield due to the measure, (2) relative change in cost due to the measure, (3) relative reduction and (4) increase in production risks associated with the measure, (5) relative climate change adaptation (i.e., benefits minus costs), (6) relative climate change mitigation, and (7) relative ecological impact (i.e., sum of change in soil and water quality and change in biodiversity richness).

#### Input data

Input data for the model included estimates based on information that we collected from the literature and other sources together with those provided by experts (see Table S1 for exact values and Annex 1 for the respective sources). The data fed into the model represent uncertainty ranges (specified by 90% confidence intervals) of the change effected by the intervention, relative to the baseline. For example, the 90% confidence interval for the increase in production costs caused by implementing a system to reuse wastewater for irrigation may range between 1 and 10%. Uncertainty ranges have been used in the past to represent uncertainty in multiple decision analysis studies (Whitney et al. 2018; Do et al. 2020; Rojas et al. 2021). We followed the general process of Applied Information Economics to improve the accuracy of our probabilistic estimates (Hubbard 2014). For each input variable, the values for the lower and upper bound of the 90% confidence interval were collated from available literature or estimated by the experts according to their experience. Since we were not able to train experts to provide model estimates through calibration training, as conducted in Do et al. (2020), we instead adjusted experts’ estimates that we considered to be too narrow resulting from overconfidence. Such an adjustment has proven useful in former decision analysis applications (Rojas et al. 2021).

#### Model implementation

We applied the general function generated from the impact pathway to all measures within a Monte Carlo simulation approach (see the *mc.simulation()* function in the decisionSupport package (Luedeling et al. 2021) for R for further details on the Monte Carlo approach). We calculated the expected outcomes for each measure based on 10,000 model iterations. For each iteration of the model, we used a set of random draws from the input dataset of estimated variable distributions (i.e., each sample is drawn from an input distribution for the respective variable, which is defined by the lower and upper bounds of its 90% confidence interval). This approach enabled us to generate a distribution of probable outcomes based on the input estimates. We compared these distributions and determined the most appropriate measures regarding the specific criteria (i.e., climate change mitigation, adaptation, and environmental impacts).

### Assumptions and simplifications

Implementing an actionable ex-ante assessment for prioritizing these 21 farm management interventions necessarily required simplification of some important relationships and output variables. In contrast to methodologies implemented in previous prioritizing approaches (Brandt et al. 2017; Mwongera et al. 2017; Andrieu et al. 2017), the mathematical function we used to represent the impact of a given intervention makes use of simple relative changes (estimated as 90% confidence intervals) without a complex quantification procedure. We identified key variables representing a potentially causal relationship between the measure and the outcome (e.g., biodiversity richness in the case of ecological impact) but did not describe underlying parameters (e.g., number of bird species in the case of biodiversity richness). Due to the complexity of assessing the impacts of the measures on expected outcomes, we did not include climate change mitigation and ecological impact for some interventions.

While climate change adaptation can be estimated through complex methodologies that include “cost effectiveness” analysis (CEA) and “multi-criteria” analysis (MCA; Nigussie et al. (2018)), we followed a “cost benefit” concept as implemented in Zhu et al. (2016) to assess adaptation options for wine production in Italy. We preferred the simpler approach over CEA and MCA due to the number and complexity of measures that we assessed as well as our general objective of generating quick, actionable decision support for difficult and risky certification schemes. We advise, however, that more complex frameworks may still be required when assessing the most promising farm interventions identified in our assessment.

### Data processing, model implementation, and figure preparation

All data generated in this study as well as the scripts developed for model implementation and generating the figures showing numeric outputs are available in a public repository (https://github.com/CWWhitney/Certification_Prioritization). We used the R programming environment (R Core Team 2019) for all data processing and analysis. We performed our analysis mainly using functions from the decisionSupport (Luedeling et al. 2021) and tidyverse (Wickham et al. 2019) libraries.

## Results

### Farm management interventions and their proposed impacts on banana production

By identifying the relationships and interactions among interventions, we categorized the initial list of alternatives into five main groups (Table 1). These groups were (1) land-use diversification, (2) energy use, (3) water use, (4) plant nutrient and pest management, and (5) waste management. In the following, we summarize the results of the literature review and expert knowledge inputs for each of the five main groups of interventions.

**Table 1:** Farm management interventions considered in sustainability certification schemes for banana plantations. For a full description of each measure, please refer to the supplementary materials accompanying this study.

Main group of interventions	Description	Specific measures in group
1. Land-use diversification	Measures intended to diversify the use of land in banana plantations and compensate for the negative impacts of banana plantations on the environment.	*1.1. Buffer zone* *1.2. Conversion of low-productivity farmland (incl. unused land)*
2. Energy use	Measures intended to reduce and improve the efficiency of traditional energy systems in banana production. This also includes the transition towards renewable systems.	*2.1. Energy use plan* *2.2. Energy equipment* *2.3. Solar energy* *2.4. Other renewable energy sources*
3. Water use	Measures intended to minimize the use of water as well as maximize the efficiency of irrigation systems in banana plantations.	*3.1. Wastewater reuse* *3.2. Water reservoir* *3.3. Anti-evapotranspiration spray* *3.4. Irrigation methods* *3.5. Irrigation scheduling* *3.6. Drainage management*
4. Plant nutrient and pest management	Measures intended to minimize the use of pesticides and synthetic fertilizers, thereby protecting the soil and sustainably managing pests and diseases.	*4.1. Composting* *4.2. Nutrient management* *4.3. Integrated pest management* *4.4. Reincorporate crop residues* *4.5. Cover crops*
5. Waste management	Measures intended to reduce the environmental footprint of banana plantations through recycling, reusing, and minimizing the use of plastics in banana plantations.	*5.1. Recycling plastic* *5.2. Waste disposal plan* *5.3. Plastic reduction* *5.4. Plastic reuse*

Measures associated with land use and diversification mainly aim to improve the ecology of banana plantations. The use of buffer zones and the transformation of low-productivity farmland (incl. unused land) into areas for conservation provides ecological benefits, including the prevention of chemical runoff and drift from banana plantations and the formation of biodiversity corridors for animals and other species. To compensate for the economic costs incurred by measures in this group, farmers can implement agroforestry systems.

In banana plantations, major energy inputs are required in packing plants, for irrigation and to operate within-plantation transportation systems. Related interventions aim to reduce energy use, maximize energy use efficiency, and promote the transition towards environmentally sustainable energy generation systems. While some measures may require only small investments (e.g., energy use plan), others may incur substantial costs (i.e., solar energy). Energy-related measures can have an impact on farm adaptation to climate change. By reducing the consumption of fossil fuel, they also produce climate change mitigation benefits.

Banana plantations are highly intensive regarding water use. Measures in the water use group aim to minimize the use of water as well as implement strategies to improve the efficiency of irrigation methods. Measures related to water use can have an impact on the ecology of the farm, and they may help in adapting to climate change by improving yields.

Measures related to plant nutrient and pest management target the use of pesticides and synthetic fertilizers. Measures in this group, for example, promote the implementation of on-farm composting, cover crops, and integrated pest management strategies. Plant nutrient and pest management measures can impact farm ecology, as well as climate change adaptation and mitigation.

Waste is a main concern in banana production systems. Waste-related interventions aim to reduce the plastic footprint of banana production by implementing strategies to both reduce and reuse plastics. This group of measures can have an impact on farm ecology and climate change mitigation.

### Graphical impact pathway for main groups of interventions

**Fig. 3:**
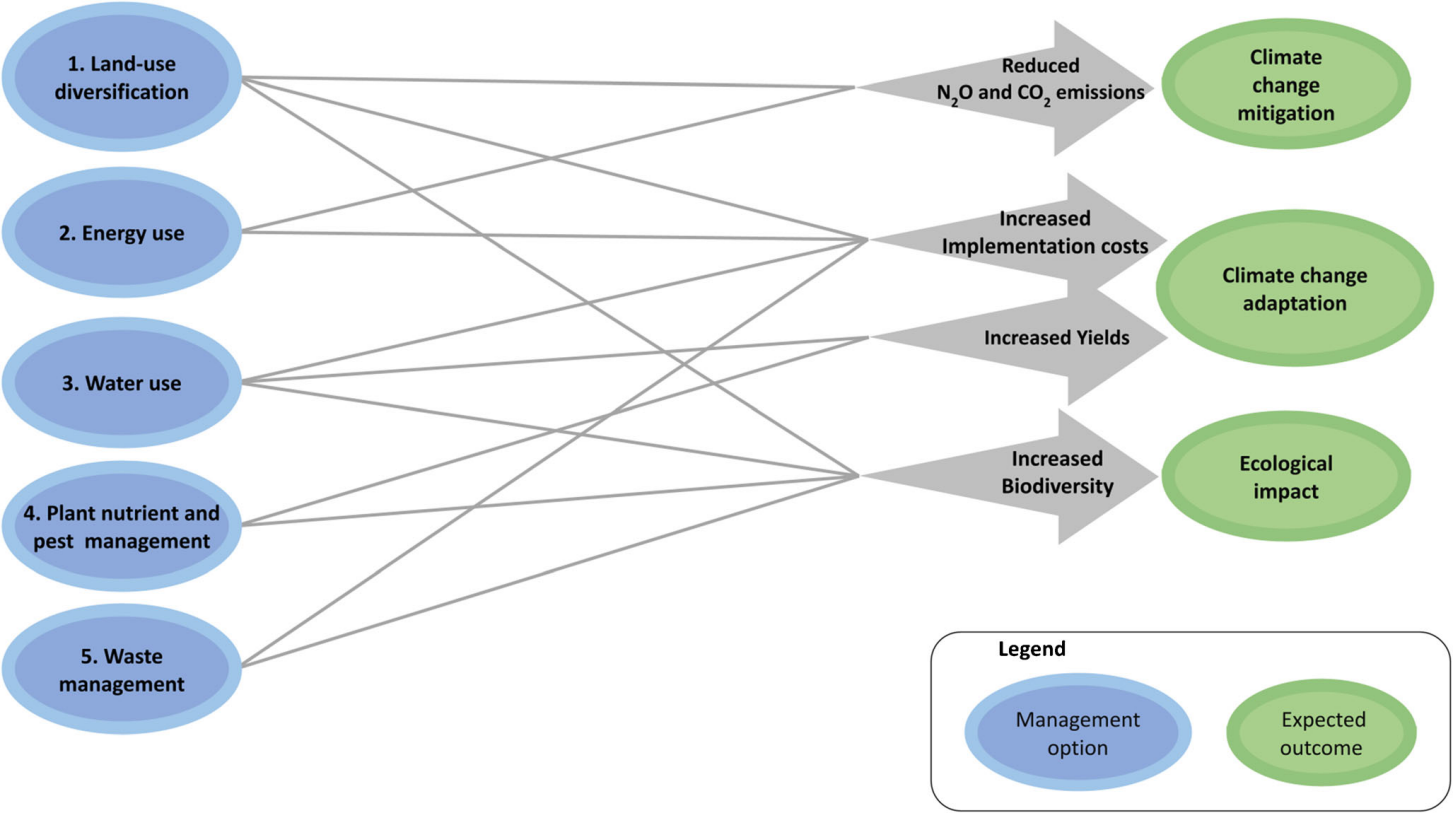
Graphical impact pathway describing the adaptation, mitigation, and ecological effects of farm management interventions proposed by GIZ to be applied in banana production systems in Latin America in order to comply with international certification schemes.

Our literature search and expert interviews allowed us to generate impact pathways regarding climate change adaptation and mitigation as well as ecological aspects (Fig. 3 and Figures S1 to S2). Regarding the effects of the measures on climate change mitigation, our approach considered global warming potential (i.e., N_2_O and CO_2_ emissions) as an indicator (Fig. 3). Relevant measures in this regard were those grouped in the “energy use” category (e.g., “energy use plan,” “solar energy,” and “other renewable energy sources”) as well as “plant nutrient and pest management” measures (e.g., “composting” and “nutrient management”). Regarding the ecological effects of the measures, we determined a number of intermediate variables (Figure S2). These variables were “groundwater fluctuation,” “potential for runoff,” “wind erosion,” “wind drift,” and “waste.” Altogether, these variables contributed to estimating the relation of the measures with the “aquatic system,” “soil system,” and “biodiversity,” which resulted in the “ecological impact” of the measures (Fig. 3).

### General impact pathways

**Fig. 4:**
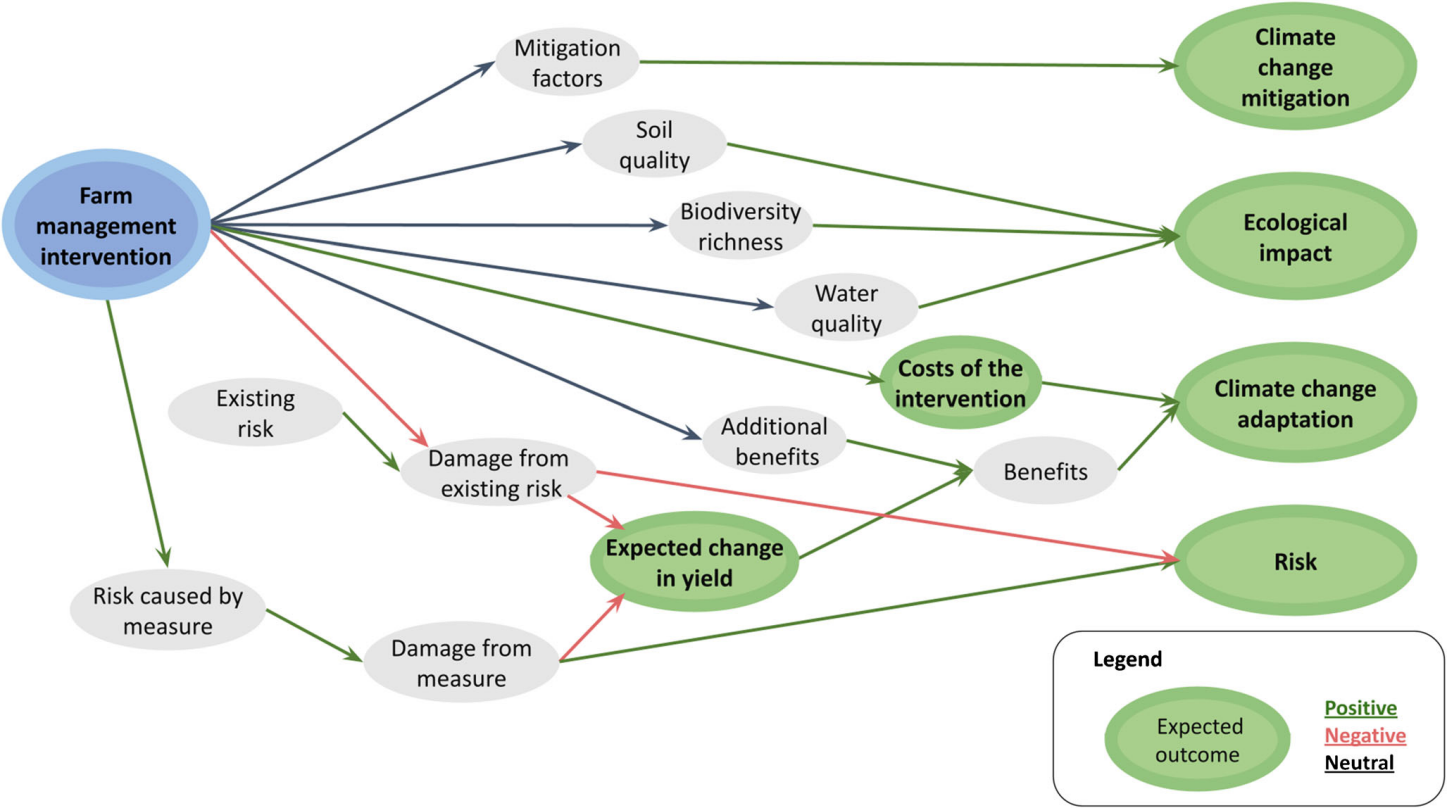
Simplified illustration of the general impact pathway implemented in the mathematical model for estimating climate change mitigation and adaptation outcomes as well as risks (increase and decrease), costs, expected change in yield, and ecological impact of farm management interventions in banana plantations.

We produced a general mathematical function (graphically represented in Fig. 4) to include the key factors that determine the potential outputs of each intervention. We identified “mitigation factors” that may reduce the emission of greenhouse gases (e.g., “composting” reduces the use of synthetic fertilizers, which release N_2_O to the atmosphere and therefore have a considerable global warming potential). These “mitigation factors,” therefore, modulate climate change mitigation outcomes. Regarding ecological impacts of the measures, we identified each measure’s impact on soil quality, biodiversity richness, and water quality (Fig. 4). In the case of climate change adaptation, we identified and estimated the cost as well as benefits of a measure and then computed the differences between them. Benefits resulted from “expected change in yield” plus “additional benefits” (e.g., lower cost incurred for synthetic fertilizer in the case of composting) generated by a given intervention. Finally, we estimated the risk (increase and decrease) associated with each measure (Fig. 4).

### Prioritized intervention options

**Fig. 5:**
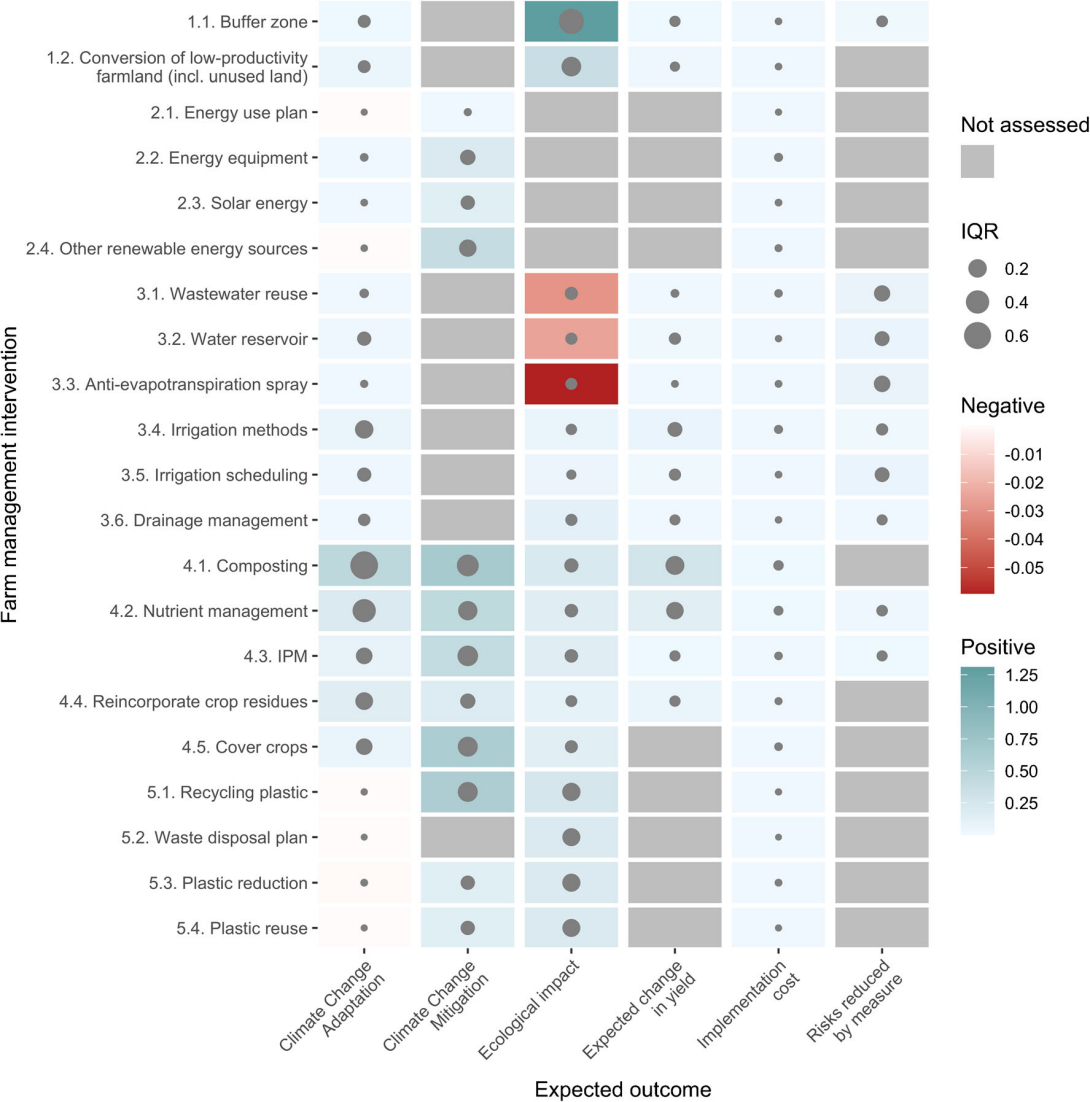
Relative farm-level effects on climate change adaptation and mitigation, ecology and production costs, change in yield and risks for 21 farm management interventions proposed for sustainable banana production systems in export-oriented banana growing regions of Latin America. In each column of the plot, we show the expected outcomes from measure implementation, whereas the proposed interventions are shown in rows. On the *y*-axis, IPM stands for integrated pest management. The color in each cell represents the likely effect (estimated as the median relative effect after 10,000 simulation runs) of the measure on the expected outcome. In gray, we show no estimated effect, whereas in blue and red we show positive and negative effects, respectively. The gray bubble inside each cell (IQR legend) represents the interquartile range, which is the difference between the third and first quartiles of the outcome distribution. We used the IQR value to express the uncertainty of our estimation of effects. Here, lower IQR values represent greater levels of confidence in the estimate.

Implementing cover crops, composting, reincorporating crop residues, integrated pest management, and nutrient management appeared to be the most relevant measures for adapting banana plantations to climate change. Although composting showed the greatest uncertainty among interventions, with climate change adaptation values ranging from −1.7% (5th quantile) to 272.0% (95th quantile), this measure appears to greatly improve farm adaptation to climate change (Fig. 5). The remaining measures in the main group “4. Plant nutrient and pest management” showed median climate change adaptation potential ranging from 7% for cover crops to 20% for nutrient management. Compared to interventions in other groups, these strategies had the highest estimated uncertainties (featuring interquartile ranges between 14% for cover crops and 40% for nutrient management). On the other hand, recycling, reducing, and reusing plastic, among others (Fig. 5), are likely to produce a slightly negative impact on farm adaptation to climate change.

Similar to the effects observed for adaptation to climate change, implementing a composting strategy in banana plantations, using cover crops, reincorporating crop residues and applying appropriate nutrient and pest management is likely to contribute to reducing the global warming potential of banana production systems (Fig. 5). Additionally, the measures related to plastic recycling and reuse as well as efficient use of energy were found to contribute to climate change mitigation.

Implementing buffer zones in banana plantations was found to generate the greatest positive effect on the ecology of the farm (Fig. 5). Our analysis also suggested that conversion of low-productivity farmland (incl. unused land) could be highly relevant for improving the environment in the plantations as well as in surrounding areas. It should be noted that measures regarding soil management (i.e., composting, cover crops, and nutrient management) were found to be relevant for improving farm ecological parameters. Similarly, measures associated with waste management (i.e., plastic reuse and waste plan) were also relevant. Water reservoirs, using wastewater and spraying anti-evapotranspiration products, might have negative effects on the ecology of the farm.

All measures generate a likely increase in production costs for farmers. Composting, nutrient management, and irrigation methods required the greatest investment by the farmers. Measures to re-incorporate crop residues and implement integrated pest management were among the least expensive.

## Discussion

Implementation of climate change adaptation and mitigation strategies in agriculture must rely on informed decision-making processes. Our decision analysis approach integrates expert and documented knowledge to provide a general overview for the prioritization of farm management interventions regarding their contribution to climate change adaptation and mitigation as well as ecological aspects. Previous studies have presented prioritization schemes for climate-smart agriculture (CSA) measures based on a consensus-driven decision support framework (“targetCSA” in Brandt et al. 2017), a four-phase prioritization framework (“CSA-PF” in Andrieu et al. 2017) or a rapid appraisal assessment (“CSA-RA” in Mwongera et al. 2017). These and other similar frameworks often include a relatively complex structure that usually assesses climatic, socio-cultural, economic, and technological characteristics of the household, farm, and community where the measures are to be implemented (Mwongera et al. 2017). The comprehensive assessments generated by such frameworks often produce results with a high degree of precision but without adequate representation of uncertainties. This makes it difficult for users to interpret assessment results for decision-making, and it incurs the risk of misrepresenting the nature of the real world. Providing certification schemes with precise but possibly wrong outcome estimates could threaten the success of certification programs, especially in the planning phase where real-world experiences are often scarce. Our prioritization framework embraces the inherent uncertainty in choosing among multiple farm management interventions that can be used in international certification schemes. Compared to the frameworks mentioned above, our decision analysis approach integrates the uncertainty around expected outcomes and allows us to provide stakeholders with likely ranges based on the inputs used to feed the model function (Whitney et al. 2018; Do et al. 2020; Ruett et al. 2020; Rojas et al. 2021).

Only a few studies report on methods and approaches that provide rapid quantitative impact analyses (Alam and Sikka 2019). Alam and Sikka (2019) demonstrated the use of a simple spreadsheet tool based on the water balance to prioritize 10 land and water interventions in two villages of Madhya Pradesh, India. Our approach offers a more participatory alternative that includes feedback from relevant stakeholders during the process of building the graphical impact pathways as well as during model implementation. By implementing a participatory approach, we attempted to mirror more complex prioritization approaches such as the “targetCSA,” “CSA-PF,” and “CSA-RA” (Brandt et al. 2017; Mwongera et al. 2017; Andrieu et al. 2017). The trade-off between framework complexity, generalization, and inclusion of relevant stakeholders needs to be negotiated in order to successfully compare multiple interventions. While our analysis allowed the comparison of all measures by using a common model structure, the mode of analysis may have oversimplified some interactions modulating the impacts of a measure on adaptation and mitigation to climate change and ecological outcomes. While we acknowledge that more complex methods (e.g., “targetCSA,” “CSA-PF,” “CSA-RA”) may be appropriate to prioritize a reduced number of specific intervention measures, we demonstrate the applicability of decision analysis approaches to implement a rapid and general assessment of numerous farm management alternatives. Along the same lines, we highlight that more complex definitions of climate change adaptation (e.g., including socio-ecological aspects) as well as more comprehensive methods for assessing this variable (e.g., “CEA” or “MCA”) may be needed in follow-up studies. Detailed decision analysis studies assessing the impacts of a specific measure on the anticipated outcomes may still be required to confidently deliver specific recommendations.

Climate change has already affected banana plantations in Latin America and other producing countries, with future global warming threatening the sustainability of cultivation of this crop. Major impacts include variations in yield (increments and declines depending on the region), shifting of growing areas (Machovina and Feeley 2013; Varma and Bebber 2019), and increased incidence of pests and diseases (Bebber 2019). Interventions that produce a positive impact on climate change adaptation are crucial to sustain banana production in Latin America. Our results suggest that farm management interventions associated with nutrient and pest management are among the most relevant to promote farm adaptation to climate change. In the case of composting, a positive impact on adaptation may result because of the low cost of inputs such as plant materials and animal manures, which are naturally produced in the plantation, and the contribution of this measure to increase banana yields (Bekunda and Woomer 1996; Ouédraogo 2001). Similarly, nutrient management practices may contribute to farm adaptation by reducing the use of chemical fertilizers (lowering production costs) without compromising yields (Israeli et al. 1985; Lobell 2007; Wairegi and van Asten 2010; Keshavan et al. 2011; Pramanik et al. 2016). Although they apply a different approach to determining climate change adaptation in their study (i.e., a multi-criteria analysis called PROMETHEE II), Nigussie et al. (2018) reported that interventions addressing soil and land management such as crop rotation and composting were the most important for farm adaptation in the Upper Blue-Nile basin in Ethiopia.

Traditional growing practices in banana plantations have contributed to climate change by emitting greenhouse gases. The carbon footprint of banana production has been estimated in different regions including Ecuador, Costa Rica, and Brazil, with values ranging between 0.46 and 1.37 kg of CO_2_ equivalent per kg of banana produced. Our results suggest that recycling plastic and implementing cover crops, nutrient management, and composting are among the most relevant measures to mitigate a farm’s global warming potential. Although the magnitude of the effect differed from our results, Andrieu et al. (2017) reported a similar positive impact of using an industrial bio-fertilizer and the production and use of on-farm compost on climate change mitigation in Malian regions (both scoring 2.0 points on a scale from −10.0 to 10.0). Comparable results regarding compost and fertilizer use on farms of Uganda and Tanzania were reported by Mwongera et al. (2017). Overall, these results concur with the findings reported by Coltro and Karaski (2019) and Iriarte (2014) who identified the intensive use of nitrogen-based fertilizers as the main driver of farm-scale GHG emissions. Along the same lines, additional carbon emission sources from agriculture include the production and acquisition of materials and the use of plastics, pesticides, and energy (burning fossil fuel and/or electricity). Accordingly, our analysis highlighted most measures within the energy use and waste management groups as positively associated with climate change mitigation. Energy-saving alternatives (e.g., insulation and efficient lighting) emerged as the most attractive group of measures for Dutch farmers when compared to the adoption of renewable energy interventions (Moerkerken et al. 2020), highlighting the importance of cost-effectiveness in determining the willingness of farmers to decide on a given measure. To facilitate the certification process, certification schemes must consider the socio-economic characteristics of targeted plantations when defining mandatory measures. Such a situation is particularly relevant in the case of smallholder farms with limited resources.

Our results suggest that the implementation of buffer zones and conversion of low-productivity farmland (incl. unused land) are likely to be the most beneficial measures regarding ecological aspects. Vegetation strips in vineyards of Central Chile, which may be seen as a type of buffer zone, were suggested to represent an important opportunity for biodiversity conservation and landscape connectivity (Díaz-Forestier et al. 2021). Regarding the contribution of these interventions to water and soil aspects, grass and forest buffer zones in Australian plantations were reported to provide ecological benefits by limiting run-off and soil erosion (McKergow et al. 2004). In addition to ecological benefits, buffer zones and the conversion of low-productivity farmland (incl. unused land) can be managed as profitable agroforestry systems, as demonstrated by Rahman et al. (2014) for agro-silvo-horticulture options in the Madhupur National Park of Bangladesh. Our results highlight potentially negative ecological outcomes regarding wastewater use, building water reservoirs, and use of anti-evapotranspiration sprays. Considering the water footprint of banana plantations, farmers and certification schemes should opt for the more ecologically friendly alternatives (e.g., irrigation methods and scheduling) to maximize the efficiency of this resource.

## Conclusions

We demonstrated the applicability of decision analysis approaches for prioritization of farm management interventions. We generated models that we were able to apply to assess the impacts that 21 interventions could have on climate change mitigation and adaption as well as the ecology of the farms. This allowed us to make recommendations to certifiers regarding the efficacy of the various options. Compared to prioritizing frameworks used in the past, our approach embraces the inherent uncertainty in the early stages of farm management intervention formulation and development. We included the participatory insights usually considered in prioritization frameworks developed to assess climate-smart agriculture measures. While more complex frameworks may provide deeper understanding on the impacts of specific management interventions, the approach we have demonstrated allows for the ex-ante comparison of several complex measures without the need for intensive data collection or long-term studies.

Our results identify measures associated with the management of nutrients (i.e., composting, nutrient management plan, cover crops) as the most relevant across the expected outcome categories (climate change mitigation and adaptation as well as ecology). Buffer zones are likely to improve the ecology of farms at relatively low implementation costs. While some interventions may appear very promising regarding their potential climate change adaptation and mitigation as well as the environmental impacts, their implementation may face social, cultural, and political challenges that are difficult to anticipate. However, traditional banana growers may be more likely to adopt farm management interventions that promise a noticeable impact on banana yields. Further studies are required to systematically determine the likelihood of implementing these or any other farm management interventions.

Future assessments should focus on a subset of interventions in order to achieve a comprehensive description of the relationships between variables modulating the expected outcomes for each measure. By adopting such an approach, decision analysis methods may prove a valid and robust framework to prioritize interventions considered by certification schemes as well as CSA frameworks. Using a validated and robust approach for prioritizing interventions is likely to support farmers and practitioners in their adaptation to the impacts of climate change.

## Supplementary information

ESM 1 (PDF 1.39 MB)

## Data Availability

Data and materials are available at https://github.com/CWWhitney/Certification_Prioritization.

## Code availability

Code is available at https://github.com/CWWhitney/Certification_Prioritization.
