# Supplementary material for: Prioritizing farm management interventions to improve climate change adaptation and mitigation outcomes—a case study for banana plantations
Source: Agron Sustain Dev. 2022 Aug 4;42(4):76. doi: 10.1007/s13593-022-00809-0 (PMC9362632; doi:10.1007/s13593-022-00809-0)
Supplement: Supplementary file 1 — (PDF 1.39 MB) [file 13593_2022_809_MOESM1_ESM.pdf]

Supplementary materials for 'Prioritizing certification interventions to improve climate change adaptation and mitigation outcomes - a case study for banana plantations'

Eduardo Fernandez[a,b], Hoa Do[a], Eike Luedeling[a], Thi Thu Giang Luu[a] and Cory Whitney[a*]

[a] Department of Horticultural Science, Institute of Crop Science and Resource Conservation (INRES), Auf dem Hügel 6, D-53121 Bonn, Germany
[b] Escuela de Agronomía, Pontificia Universidad Católica de Valparaíso, Casilla 4-D, Quillota, Chile

Introduction

In the following document, we offer supplementary information on the materials and methods as well as the results of the work 'Prioritizing certification interventions to improve climate change adaptation and mitigation outcomes - a case study for banana plantations' by Fernandez et al. (2022). The work is published in the journal Agronomy for Sustainable Development under the doi: 10.1007/s13593-022-00809-0. As mentioned in the main text, all scripts and analyses are available in a public repository hosted at: https://github.com/CWWhitney/Certification_Prioritization.

Annex 1: List of measures

After consultation with the experts and the literature we reduced the overall list of certification options to 21 interventions, categorized into 5 groups. *For each of these, the growers/producers/producer groups would be responsible for implementation.* The descriptions for each measure are provided below. We also provide the sources of information for model inputs for each measure. *Sources, where mentioned, were used in addition to experts and our own estimates.*

1. Land-use diversification

1.1. Buffer zone

Buffer zone certification measures would require that banana plantations include vegetative buffers at the edges of cropped fields. In this measure growers are required to maintain existing riparian buffer zones around aquatic ecosystems, bodies of water and watershed recharge areas and between production and areas of high conservation value, either protected or not. Pesticides, hazardous chemicals and fertilizers are not applied. The buffer zones could be covered with grass, shrubs, trees or a mix of vegetation (McKergow et al. 2004). Buffer zones provide mainly ecological benefits such as preventing chemical runoff and drift (McKergow et al. 2004) and act as biodiversity corridors (Ducros and Joyce 2003). The implementation of buffer zones may be costly and also have trade-offs with the banana yield (area sacrificed to buffer zones). The practice of agroforestry in the buffer areas may provide a successful management strategy for both environmental and economic benefits (Rahman et al. 2014).

Input data (i.e. 90% confidence intervals) for all variables associated with this measure were obtained from various sources including Collentine et al. (2015), Ducros and Joyce (2003), Melo and Wolf (2005), McVittie et al. (2015), Muscutt et al. (1993) and Zhu, Yang, and Zhang (2021). We updated these values using knowledge from experts as well as our own judgement when we considered the intervals to be too narrow.

*Dr. Cory Whitney (cory.whitney@uni-bonn.de)

1.2. Conversion of low-productivity farmland (incl. unused land)

In this certification measure, banana growers would convert unproductive sites into conservation areas where viable. They would develop a map that includes natural ecosystems and agroforestry canopy cover or border plantings with estimated vegetation coverage and estimated percentage of native species composition and progressively increase or restore native vegetation adjacent to aquatic ecosystems, farmed areas of marginal productivity and around housing and infrastructure. This could include live fences, shade trees and permanent agroforestry systems. Our models assumed that the unused land areas are likely to be a rather small portion of the total plantation land. There will be economic benefits from other harvest (e.g. from the agroforestry system) and ecological benefits such as increasing farm biodiversity.

Input data (i.e. 90% confidence intervals) for all variables associated with this measure were mainly obtained from Vallejo-Chaverri et al. (2018). We updated these values using knowledge from experts as well as our own judgement when we considered the intervals to be too narrow.

2. Energy use

2.1. Energy use plan

With this measure banana producers would be required to keep track of the energy consumption and explore the options to reduce environmental impacts and costs associated with the non-renewable energy use. Within the banana production system, energy sources such as fossil fuel and electricity are mainly used in packing plants (e.g. lighting, water supply and conveyor belt) and to extract water for irrigation and to operate within-plantation transportation systems.

Input data (i.e. 90% confidence intervals) for all variables associated with this measure were obtained from various sources including Păunescu and Blid (2016) and Vallejo-Chaverri et al. (2018). We updated these values using knowledge from experts as well as our own judgement when we considered the intervals to be too narrow.

2.2. Energy equipment

With this measure banana growers would select and invest in energy-efficient equipment where possible and maintain it for optimum energy consumption. This measure could help to reduce the energy consumption in the production system and lower energy costs. The measure could also reduce overall emissions due to lower fossil fuel combustion.

Input data (i.e. 90% confidence intervals) for all variables associated with this measure were obtained from various sources including Akcaoz (2011) and Lin, Lin, and Peng (2019). We updated these values using knowledge from experts as well as our own judgement when we considered the intervals to be too narrow.

2.3. Solar energy

This certification measure requires that producers reduce the use of non-renewable energies and offset or replace them with solar energy. According to experts, solar may already be feasible (cost effective) in some banana plantations in Latin America but is not yet a widely available option.

Input data (i.e. 90% confidence intervals) for all variables associated with this measure were obtained from various sources including Akcaoz (2011), Jacobs et al. (2013), Pogson, Hastings, and Smith (2013), Sarath, Uma, and Kumar (2017), Viviescas et al. (2019) and https://www.ecowatch.com/solar/solar-panel-payback. We updated these values using knowledge from experts as well as our own judgement when we considered the intervals to be too narrow.

2.4. Other renewable energy sources

This certification measure requires that producers reduce the use of non-renewable energies and offset or replace them with biomass energy. In this certification measure organic wastes from banana production are used for generating power on the plantation. The biomass used for energy generation can be costly and resource intensive (water, soil, synthesized inputs, energy etc.). Efficient technologies to make this practicable may not be widely available (i.e. cost effectiveness may be an issue).

Input data (i.e. 90% confidence intervals) for all variables associated with this measure were mainly obtained from Tock et al. (2010). We updated these values using knowledge from experts as well as our own judgement when we considered the intervals to be too narrow.

3. Water use

3.1. Wastewater reuse

Packing bananas, which uses a lot of water, is done daily during the harvest season. This certification measure requires that producers collect and re-use this water for irrigation. However, the wastewater from packing plants is prone to risks of salinity, phytotoxicity and other contaminants which can affect banana plants, soil and water ecosystems. In addition, wastewater from banana processing contains latexes that might impact soils and the banana fields. Proper treatment before using for irrigation is needed. The measure may reduce the risk of water shortage for irrigation but may incur additional costs for water treatment.

Input data (i.e. 90% confidence intervals) for all variables associated with this measure were obtained from various sources including Russo and Hernández (1995) and van Asten, Fermont, and Taulya (2011). We updated these values using knowledge from experts as well as our own judgement when we considered the intervals to be too narrow.

3.2. Water reservoir

This certification measure requires the construction of reservoirs to collect rainwater and store water for dry periods. This can help to reduce risks of water shortage for production and at the same time reduce the impact of high rain intensity (i.e. surface runoff and waterlogging) during heavy rain events. The water storage also reduces ground water withdrawal, which may have a positive impact on aquatic ecosystems. The reservoirs can be trenches that are dug along plantation contours to keep water around the cultivated areas. The costs involved could be mainly labor and basic construction materials for digging and maintaining the trenches. The trade-off could be less land available for banana production.

Input data (i.e. 90% confidence intervals) for all variables associated with this measure were mainly obtained from Mugerwa (2007) and http://www.agritech.tnau.ac.in/expert_system/banana/irrigationmanagement.html#5. We updated these values using knowledge from experts as well as our own judgement when we considered the intervals to be too narrow.

3.3. Anti-evapotranspiration spray

This certification measure requires the use of organic anti-evapotranspiration substances to reduce water evapotranspiration and increase water use efficiency of bananas. The use of anti-transpirant in combination with appropriate irrigated regimes can reduce the total amount of irrigated water during the growing season (El-Kader 2006). However, in high rainfall regions the use of organic foliar spray against evapotranspiration may be less relevant than in low rainfall regions. It could suppress banana growth and development by reducing photosynthesis. Methods such as mulching and ground cover may be more effective in managing evaporation from the soil surface.

Input data (i.e. 90% confidence intervals) for all variables associated with this measure were mainly obtained from El-Kader (2006) and Gawad (2014). We updated these values using knowledge from experts as well as our own judgement when we considered the intervals to be too narrow.

3.4. Irrigation methods

With this certification measure banana growers would improve irrigation methods. Improved irrigation can increase both water-use efficiency and yield. According to experts, under canopy single- and series-sprinkler irrigation systems are the most common irrigation techniques for large scale banana production. Experts agree that this is a highly efficient method. Furrow irrigation, flood irrigation are common but are considered low efficiency. Drip irrigation is also applied on some plantations and could perform well in semi-arid areas where availability of water is low.

Input data (i.e. 90% confidence intervals) for all variables associated with this measure were obtained from various sources including de Oliveira et al. (2009), N. Panigrahi et al. (2021), Pawar, Dingre, and Bhoi (2017) and Pramanik

and Patra (2016). We updated these values using knowledge from experts as well as our own judgement when we considered the intervals to be too narrow.

3.5. Irrigation scheduling

Irrigation scheduling requires the calculation of crop water requirement, crop water demand at different growth stages, soil moisture, evapotranspiration rate, among other water use factors (P. Panigrahi et al. 2019). Certification requirements for irrigation scheduling could provide synchrony of water needed and the quantity of water supplied which, in turn, could enhance irrigation efficiency and reduce water waste (Israeli, Hagin, and Katz 1985; N. Panigrahi et al. 2021).

Input data (i.e. 90% confidence intervals) for all variables associated with this measure were obtained from various sources including de Oliveira et al. (2009), Minhas et al. (2020) and N. Panigrahi et al. (2021). We updated these values using knowledge from experts as well as our own judgement when we considered the intervals to be too narrow.

3.6. Drainage management

With this certification measure managers would be required to design drainage systems based on the biophysical characteristics of the plantation such as soil type and structure, water system, slope and ground cover to improve farm drainage capacity. They would also identify erosion prone areas, areas with high risk of flooding and those with poor drainage conditions. According to experts, open systems with water channels along the banana plots are common. These perform sufficiently to avoid waterlogging. A good drainage system will help to reduce the risk of waterlogging which may be critical in high rainfall regions.

Input data (i.e. 90% confidence intervals) for all variables associated with this measure were mainly obtained from Vallejo-Chaverri et al. (2018). We updated these values using knowledge from experts as well as our own judgement when we considered the intervals to be too narrow.

4. Plant nutrient and pest management

4.1. Composting

With this certification measure banana growers would make compost from farm plant residues and use compost and green fertilizers as a source of plant nutrients. This may reduce the cost for chemical fertilizers. Farmers can combine compost with other sources of nutrients, which has been proved to contribute to increase the yield of crops (Bekunda and Woomer 1996; Ouédraogo 2001). Application of compost can increase soil fertility and microbial activities as well as enhance the water holding capacity of sandy soil. Composting requires low cost of inputs such as plant materials, animal manures that can be found around the plantation. However, the practice may require more labor in the process of making compost.

Input data (i.e. 90% confidence intervals) for all variables associated with this measure were obtained from various sources including Kukulies et al. (2014), Meya et al. (2020) and Wairegi and Asten (2010). We updated these values using knowledge from experts as well as our own judgement when we considered the intervals to be too narrow.

4.2. Nutrient management

This measure would require farmers to apply nutrient management practices based on assessments of crop needs, regular monitoring of soil fertility and crop nutrient status, or recommendations from local agronomic experts. Regular soil tests and leaf tests including macro- and micro-nutrients and organic matter would be carried out frequently. Management practices such as choosing appropriate nutrient doses, forms and sources as well as deciding on the right time and method of application may help farmers to reduce chemical fertilizer used without compromising the yield (Israeli, Hagin, and Katz 1985; Keshavan, Kavino, and Ponnuswami 2011; Lobell 2007; Wairegi and Asten 2010). The proper management of fertilizer application, especially nitrogen, could mitigate greenhouse gas (i.e. nitrous oxide - N2O and nitric oxide - NO) emission (Masters 2019; Rowlings et al. 2013; Veldkamp and Keller 1997). The synchronization of fertilizer application and crop demand could minimize chemical residues in drinking water and aquatic ecosystems (Henriques et al. 1997; Stover 1986; Svensson et al. 2018).

Input data (i.e. 90% confidence intervals) for all variables associated with this measure were obtained from various sources including Aryal et al. (2012), Bellamy (2013), Coltro and Karaski (2019), Iriarte (2014), Masters (2019), Meya et al. (2020), @ Rowlings2013Influence, Strobl and Mohan (2020) and Svanes and Aronsson (2013). We updated these values using knowledge from experts as well as our own judgement when we considered the intervals to be too narrow.

4.3. Integrated Pest Management (IPM)

This measure requires that banana growers to develop an integrated pest management (IPM) plan and follow regulations on sprays of pesticides. They would implement various IPM activities that could reduce the incidence and intensity of pest attacks, and thereby reduce the need for chemical intervention. Growers would also take part in training on integrated pest management including monitoring of pests and diseases, alternative ways to control pests and diseases, preventive measures against pests and diseases, measures to avoid buildup of pest and disease resistance to pesticides. They keep a list of the pesticides with names of active ingredients, crops on which the pesticides were used and the targeted pests. The implementation could help to reduce chemicals used which will decrease production costs and emissions from chemical manufacturing, as well as avoid economic losses due to pest incidence. The production systems with reduced pesticide application could also have less negative impacts on human and wildlife (e.g. acute toxicity) (Henriques et al. 1997) and local biodiversity.

Input data (i.e. 90% confidence intervals) for all variables associated with this measure were obtained from various sources including Barraza et al. (2011), Barraza et al. (2020), Blazy et al. (2009), Castillo et al. (2006), Chaves, Shea, and Cope (2007) and Côte et al. (2009). We updated these values using knowledge from experts as well as our own judgement when we considered the intervals to be too narrow.

4.4. Reincorporate crop residues

With this certification measure banana growers would be required to use organic waste from their farm production for mulching. The banana residue would be retained in the field. This could also contribute to reducing organic waste from the plantation and increase soil cover to prevent runoff. Mulching may also to increase banana yields as it was the case with mulching, in combination with mineral fertilizers, in banana plantations in Uganda (Wairegi and Asten 2010).

Input data (i.e. 90% confidence intervals) for all variables associated with this measure were mainly obtained from Tursun et al. (2018). We updated these values using knowledge from experts as well as our own judgement when we considered the intervals to be too narrow.

4.5. Cover crops

With this measure banana farmers would plant cover crops to avoid bare soils, reduce erosion and weed infestation. The ground cover could reduce nutrient losses from leaching (cover crop as catch crop) and mitigate greenhouse gas emission (Abdalla et al. 2019; Lavigne et al. 2012). However, planting cover crops incurs costs for establishing and maintaining the vegetation. There is also a chance that cover crops will compete for resources with cash crops which may affect the yield (Abdalla et al. 2019; Lavigne et al. 2012).

Input data (i.e. 90% confidence intervals) for all variables associated with this measure were obtained from various sources including Abdalla et al. (2019), Blazy et al. (2009), Johns (1994), Kukulies et al. (2014), Quaresma, Oliveira, and Silva (2017), Tursun et al. (2018) and Vallejo-Chaverri et al. (2018). We updated these values using knowledge from experts as well as our own judgement when we considered the intervals to be too narrow.

5. Waste management

5.1. Recycling plastic

This certification measure would require banana farmers to collect plastic used in the farm and send it to plastic recyclers. The practice may incur small labor cost for gathering and compacting used plastic materials. Plastic recycling will mainly have ecological benefits for both terrestrial and aquatic ecosystems.

Input data (i.e. 90% confidence intervals) for all variables associated with this measure were mainly obtained from Russo and Hernández (1995) and Svanes and Aronsson (2013). We updated these values using knowledge from experts as well as our own judgement when we considered the intervals to be too narrow.

5.2. Waste disposal plan

The commercial banana plantations face a challenge managing their waste, particularly plastic waste used to protect the plant during its growing period and solid waste used in the packing plant for post-harvest (Russo and Hernández 1995). The banana producer therefore needs a concrete management plan for proper disposal of those undesired by-products. This certification measure would require farmers to calculate and record types and amounts of waste from different units of the production process for identifying the potential measures for waste treatments.

Input data (i.e. 90% confidence intervals) for all variables associated with this measure were obtained from various sources including Melo and Wolf (2005), Russo and Hernández (1995) and Vallejo-Chaverri et al. (2018). We updated these values using knowledge from experts as well as our own judgement when we considered the intervals to be too narrow.

5.3. Plastic reduction

This certification measure would require banana growers to reduce plastic use by using a continuous polyethylene tube instead of the standard pre-cut impregnated plastic bags to protect banana bunches. It is possible to minimize plastic use by fitting the tube to the exact length of the bunches. This method can increase labor costs when using the replacement plastics. However, it also helps reduce plastics purchased, thus reducing production costs, plastics produced as well as environmental impacts on land, water bodies and human habitats.

Input data (i.e. 90% confidence intervals) for all variables associated with this measure were mainly obtained from Russo and Hernández (1995). We updated these values using knowledge from experts as well as our own judgement when we considered the intervals to be too narrow.

5.4. Plastic re-use

This certification measure would require banana farmers to re-use plastics, such as the protecting bags and plastic twine for holding up banana plants. The measure incurs an increased labor cost to collect and treat the bags again before reuse. Plastic reuse may reduce production costs and reduce plastic waste, which will contribute to both economic and environmental benefits.

Input data (i.e. 90% confidence intervals) for all variables associated with this measure were mainly obtained from Russo and Hernández (1995). We updated these values using knowledge from experts as well as our own judgement when we considered the intervals to be too narrow.

Table S1: Model inputs

We generated a table of confidence estimates (90%) for use in the decision model. Most variable values are described as a percentage difference from a baseline (in decimals). Others, such as coefficient of variation (coeff. Variation) and ecological values are described as integers.

Table S1: Estimates of inputs provided to the decision model

variable	lower	upper	label
var_CV	5.0000	20.000	coeff. Variation
discount_rate	1.0000	5.000	Discount rate (%)
n_years	10.0000	10.000	Duration of simulation (years)
prior_market_price	0.1000	0.500	Market prices for banana (USD/kg)
prior_yield	20000.0000	60000.000	Baseline yield in a normal season without any implementation of any measur(kg/ha/yr)
prior_cost	2000.0000	10000.000	Prior production costs banana production (USD/ha/yr)
base_diversify_cost	10.0000	300.000	Prior for the normal costs related to diversifying land use (USD/yr)
wind_event_risk	0.0500	0.300	Risk of wind event (%)
normal_wind_damage	0.1000	0.500	Yield lost to wind in a normal year (%)
reduction_wind_damage_buffer	0.1000	0.800	Wind damage avoided through measure implementation (%)
yield_lost_for_buffer	0.0200	0.080	Relative reduction in yield due to measure implementation (%)
cost_buffer	0.0100	0.050	Relative increase in baseline cost due to measure implementation
soil_quality_buffer	10.0000	80.000	Relative impact of the measure on soil quality
water_quality_buffer	20.0000	80.000	Relative impact of the measure on water quality
biodiv_richness_buffer	5.0000	80.000	Relative impact of the measure on biodiversity richness
yield_conversion	0.0100	0.050	Relative increase in yield due to measure implementation (%)
added_benefit_conversion	0.0100	0.050	Relative added benefit due to the measure implementation (%)
cost_conversion	0.0100	0.050	Relative increase in baseline cost due to measure implementation
soil_quality_conversion	5.0000	10.000	Relative impact of the measure on soil quality
biodiv_richness_conversion	5.0000	60.000	Relative impact of the measure on biodiversity richness
base_energy_cost	20.0000	1000.000	Prior for the normal costs related to energy use and management (USD/yr)
cost_energy_use_plan	0.0010	0.005	Relative increase in baseline cost due to measure implementation
energy_saved_use_plan	0.0010	0.005	Relative energy saved due to measure implementation
increase_efficiency_energy_use_plan	0.0010	0.005	Relative increase in energy efficiency due to measure implementation

variable	lower	upper	label
reduced_fossil_fuel_consumption_energy_use_plan	0.0010	0.005	Relative fossil fuel consumption saved due to measure implementation
cost_energy_equipment	0.0100	0.200	Relative increase in baseline cost due to measure implementation
energy_saved_energy_equipment	0.0200	0.200	Relative energy saved due to measure implementation
increase_efficiency_energy_equipment	0.0200	0.200	Relative increase in energy efficiency due to measure implementation
reduced_fossil_fuel_consumption_energy_equipment	0.0200	0.200	Relative fossil fuel consumption saved due to measure implementation
cost_solar_energy	0.0010	0.040	Relative increase in baseline cost due to measure implementation
energy_saved_solar_energy	0.0100	0.040	Relative energy saved due to measure implementation
increase_efficiency_solar_energy	0.0000	0.000	Relative increase in energy efficiency due to measure implementation
reduced_fossil_fuel_consumption_solar_energy	0.0500	0.250	Relative fossil fuel consumption saved due to measure implementation
cost_other_energy	0.0100	0.040	Relative increase in baseline cost due to measure implementation
energy_saved_other_energy	0.0100	0.040	Relative energy saved due to measure implementation
increase_efficiency_other_energy	0.0000	0.000	Relative increase in energy efficiency due to measure implementation
reduced_fossil_fuel_consumption_other_energy	0.2000	0.600	Relative fossil fuel consumption saved due to measure implementation
base_water_cost	20.0000	1000.000	Prior for the normal costs related to water use and management (USD/yr)
cost_waste_water_use	0.0100	0.100	Relative increase in baseline cost due to measure implementation
salinity_risk	0.0500	0.900	Risk of having salinity issues in water
dry_spells_risk	0.0200	0.500	Risk of dry spells
normal_dry_spell_damage	0.2000	0.800	Yield lost to dry spells in a normal year (%)
waste_water_deficit_reduction	0.0100	0.070	Relative decrease in water deficit due to measure implementation
normal_salinity_damage	0.0015	0.070	Normal salinity damage without negative impact of waste water
waste_water_salinity_damage	0.0100	0.100	Yield lost to salinity damage from waste water
soil_quality_waste_water	-10.0000	-1.000	Relative impact of the measure on soil quality
water_quality_waste_water	-5.0000	5.000	Relative impact of the measure on water quality
biodiv_richness_waste_water	0.1000	5.000	Relative impact of the measure on biodiversity richness
cost_water_reservoir	0.0010	0.010	Relative increase in baseline cost due to measure implementation
reservoir_water_deficit_reduction	0.1000	0.500	Relative decrease in water deficit due to measure implementation

variable	lower	upper	label
water_quality_water_reservoir	1.0000	5.000	Relative impact of the measure on water quality (reduced groundwater fluctuation)
biodiv_richness_water_reservoir	-10.0000	-1.000	Relative impact of the measure on biodiversity richness
cost_a_evap_trans_spray	0.0010	0.050	Relative increase in baseline cost due to measure implementation
a_evap_trans_spray_water_deficit_reduction	0.0010	0.020	Relative decrease in water deficit due to measure implementation
water_quality_a_evap_trans_spray	-1.0000	0.100	Relative impact of the measure on water quality (reduced groundwater fluctuation)
biodiv_richness_a_evap_trans_spray	-10.0000	-1.000	Relative impact of the measure on biodiversity richness
cost_irrigation_methods	0.0100	0.200	Relative increase in baseline cost due to measure implementation
irrigation_methods_water_deficit_reduction	0.5000	0.900	Relative decrease in water deficit due to measure implementation
soil_quality_irrigation_methods	0.1000	5.000	Relative impact of the measure on soil quality
water_quality_irrigation_methods	0.1000	2.000	Relative impact of the measure on water quality
biodiv_richness_irrigation_methods	0.1000	5.000	Relative impact of the measure on biodiversity richness
cost_irrigation_scheduling	0.0010	0.030	Relative increase in baseline cost due to measure implementation
irrigation_scheduling_water_deficit_reduction	0.1000	0.500	Relative decrease in water deficit due to measure implementation
soil_quality_irrigation_scheduling	0.1000	2.000	Relative impact of the measure on soil quality
water_quality_irrigation_scheduling	0.1000	3.000	Relative impact of the measure on water quality
biodiv_richness_irrigation_scheduling	0.1000	2.000	Relative impact of the measure on biodiversity richness
cost_drainage_mgmt	0.0010	0.020	Relative increase in baseline cost due to measure implementation
reduction_waterlog_drainage_mgmt	0.2000	0.800	Relative reduction in waterlogging due to measure implementation
flood_event_risk	0.0200	0.500	Risk of flood events
normal_waterlog_damage	0.0500	0.300	Yield lost to waterlog in a normal year (%)
soil_quality_drainage_mgmt	2.0000	7.000	Relative impact of the measure on soil quality
water_quality_drainage_mgmt	0.1000	2.000	Relative impact of the measure on water quality
biodiv_richness_drainage_mgmt	2.0000	10.000	Relative impact of the measure on biodiversity richness
base_chemical_cost	400.0000	2000.000	Prior for the normal costs related to chemical use (USD/ha/yr)
cost_composting	0.0500	0.150	Relative increase in baseline cost due to measure implementation
yield_increase_composting	0.0500	0.550	Relative increase in yield due to measure implementation
fertilizer_reduction_composting	0.1000	0.750	Relative reduction in fertilizer use due to measure implementation

variable	lower	upper	label
reduced_fertilizer_production_composting	0.1000	0.750	Relative reduction in fertilizer production due to measure implementation (farm-level responsibility of the CO2 emissions of the production)
reduced_chemical_residue_composting	0.0500	0.500	Relative reduction in chemical residues (and N20 emissions) due to measure implementation
soil_quality_composting	1.0000	10.000	Relative impact of the measure on soil quality
water_quality_composting	1.0000	7.000	Relative impact of the measure on water quality
biodiv_richness_composting	5.0000	20.000	Relative impact of the measure on biodiversity richness
cost_nutrient_mgmt	0.0500	0.100	Relative increase in baseline cost due to measure implementation
yield_increase_nutrient_mgmt	0.0100	0.400	Relative increase in yield due to measure implementation
fertilizer_reduction_nutrient_mgmt	0.0100	0.250	Relative reduction in fertilizer use due to measure implementation
pest_outbreak_risk	0.1000	0.300	Risk of pest and disease outbreak (%)
normal_damage_pests	0.1000	0.400	Yield lost to pests and disease in a normal year (%)
reduction_damage_pest_nutrient_mgmt	0.1000	0.600	Relative reduction in pest and disease damage due to measure implementation
reduced_chemical_residue_nutrient_mgmt	0.1000	0.600	Relative reduction in chemical residues (and N20 emissions) due to measure implementation
reduced_fertilizer_production_nutrient_mgmt	0.0100	0.250	Relative reduction in fertilizer production due to measure implementation (farm-level responsibility of the CO2 emissions of the production)
soil_quality_nutrient_mgmt	1.0000	5.000	Relative impact of the measure on soil quality
water_quality_nutrient_mgmt	1.0000	7.000	Relative impact of the measure on water quality
biodiv_richness_nutrient_mgmt	1.0000	15.000	Relative impact of the measure on biodiversity richness
cost_ipm_practice	0.0010	0.050	Relative increase in baseline cost due to measure implementation
pesticide_reduction_ipm_practice	0.0500	0.800	Relative reduction in pesticide use due to measure implementation
reduction_damage_ipm_practice	0.2500	0.750	Relative reduction in pest and disease outbreak damage due to measure implementation
reduced_pesticide_production_ipm_practice	0.1000	0.750	Relative reduction in pesticide production due to measure implementation (farm-level responsibility of the CO2 emissions of the production)
soil_quality_ipm_practice	0.0000	3.000	Relative impact of the measure on soil quality
water_quality_ipm_practice	2.0000	9.000	Relative impact of the measure on water quality

variable	lower	upper	label
biodiv_richness_ipm_practice	1.0000	15.000	Relative impact of the measure on biodiversity richness
yield_reincorporation	0.0300	0.100	Relative increase in yield due to measure implementation
cost_reincorporation	0.0010	0.020	Relative increase in baseline cost due to measure implementation
herbicide_reduction_reincorporation	0.1000	0.500	Relative reduction in herbicide use due to measure implementation
fertilizer_reduction_reincorporation	0.0100	0.250	Relative reduction in fertilizer use due to measure implementation
reduced_herbicide_production_reincorporation	0.0500	0.300	Relative reduction in herbicide production due to measure implementation (farm-level responsibility of the CO2 emissions of the production)
soil_quality_reincorporation	1.0000	8.000	Relative impact of the measure on soil quality
water_quality_reincorporation	1.0000	5.000	Relative impact of the measure on water quality
biodiv_richness_reincorporation	1.0000	5.000	Relative impact of the measure on biodiversity richness
herbicide_reduction_cover_crop	0.3000	0.900	Relative reduction in herbicide use due to measure implementation
yield_reduction_cover_crop	0.0010	0.050	Relative reduction in yield due to measure implementation
cost_cover_crop	0.0010	0.050	Relative increase in baseline cost due to measure implementation
reduced_herbicide_production_cover_crop	0.3000	0.900	Relative reduction in herbicide production due to measure implementation (farm-level responsibility of the CO2 emissions of the production)
competition_risk	0.0010	0.010	Risk of cover crop competion with banana for resources (%)
normal_competition_damage	0.0010	0.010	Yield lost to competition for resources with other plants in a normal year (%)
increased_damage_by_competition	0.0010	0.010	Yield lost to crop competition due to measure implementation
soil_quality_cover_crop	1.0000	5.000	Relative impact of the measure on soil quality
water_quality_cover_crop	1.0000	10.000	Relative impact of the measure on water quality
biodiv_richness_cover_crop	1.0000	10.000	Relative impact of the measure on biodiversity richness
base_plastic_cost	5.0000	50.000	Prior for the normal costs related to plastics and recycling (USD/yr)
recycling_cost	0.0100	0.100	Relative increase in baseline cost due to measure implementation
reduced_plastic_production_recycling	0.3000	0.900	Relative reduction in plastic production due to measure implementation (farm-level responsibility of the CO2 emissions of the production)
biodiv_richness_recycling	5.0000	50.000	Relative impact of the measure on biodiversity richness

variable	lower	upper	label
waste_plan_cost	0.0010	0.005	Relative increase in baseline cost due to measure implementation
biodiv_richness_waste_plan	3.0000	45.000	Relative impact of the measure on biodiversity richness
costs_plastic_wrapping_time	0.0500	0.500	Relative increase in baseline cost due to measure implementation
savings_reduced_plastic	0.0100	0.050	Relative decrease in baseline cost due to measure implementation (%)
reduced_plastic_production_replacement	0.0500	0.250	Relative reduction in plastic production due to measure implementation (farm-level responsibility of the CO2 emissions of the production)
biodiv_richness_reduced_plastic	3.0000	45.000	Relative impact of the measure on biodiversity richness
costs_plastic_reuse	0.0100	0.050	Relative increase in baseline cost due to measure implementation
savings_plastic_reuse	0.0100	0.050	Relative decrease in baseline cost due to measure implementation (%)
reduced_plastic_production_plastic_reuse	0.0500	0.250	Relative reduction in plastic production due to measure implementation (farm-level responsibility of the CO2 emissions of the production)
biodiv_richness_reuse_plastic	3.0000	45.000	Relative impact of the measure on biodiversity richness

Annex 2: Model function

We developed a general function that estimates costs, benefits, risk reduction and risk increase, adaptation and mitigation to climate change, and the ecological impact of any certification measure (see `certification_impact.R` in https://github.com/CWWhitney/Certification_Prioritization). This allowed us to obtain a common output structure independent of the certification measure evaluated. The simulation was run to represent 10 years of a typical banana production system.

Later, we applied this function to all certification measures using the specific information we gathered for each.The ultimate aim was to get a list of the measures that influence adaptation, mitigation and environmental outcomes (see `return()` list at the end of the `certification_measures_function.R` in https://github.com/CWWhitney/Certification_Prioritization).

After coding the impact pathways we performed a Monte Carlo simulation with the `mcSimulation()` function from `decisionSupport` (Luedeling et al. 2022). This function generates a distribution representing the desired outputs (see `return()` function above) by calculating random draws in our defined `certification_measures_function()`. Inside this simulation we use a generalized function called `certification_impact()` to establish the possible impacts of each measure.

```
source("certification_measures_function.R")
certification_measures_simulation <- mcSimulation(
  estimate = estimate_read_csv("certification_measures_input_table.csv"),
  model_function = certification_measures_function,
  numberOfModelRuns = 1e4, #10000 runs
  functionSyntax = "plainNames"
)
```

Supplementary figures

Figure S1

In Fig. S1 we show the detailed impact pathway representing the potential underlying relationships between the certification measures and the farm productivity (i.e. adaptation aspect). For the measures in the Energy group, we estimated a decline in energy consumption as well as a positive impact on implementation costs. The relationships within the remaining groups were more complex. For instance, our model suggests salinity as a potential driver for banana yields in case wastewater is used for irrigation (Fig. S1). Similar intermediate variables affecting the productivity of the farm can be identified in our impact pathway.

Figure S2

In Fig. S2 we show the detailed impact pathway representing the potential underlying relationships between the certification measures and global warming potential and ecological aspects. For global warming, we identified fossil fuel consumption as a driver of greenhouse gas emission (Fig. S2). In the case of ecological aspects, we identified a number of variables modulating the impact on the environment. Among them, soil salinity, water supply capacity, overland flow, organic matter and fertilizer and pesticide use could be key determinants for the measures' impacts in this regard (Fig. S2).

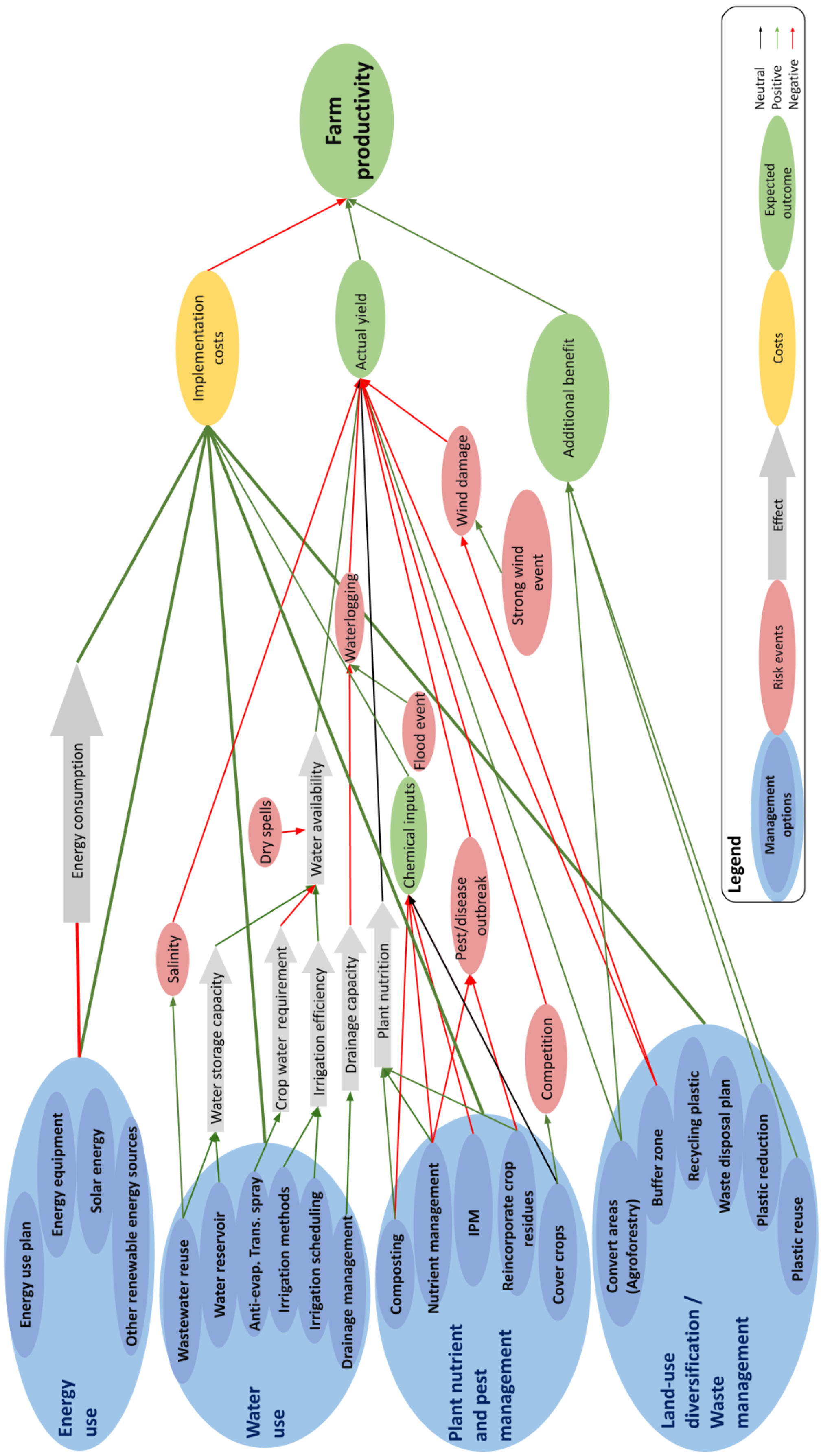

Figure S1: Detailed impact pathway representing the potential underlying variables modulating the impact of 21 certification measures on farm adaptation to climate change

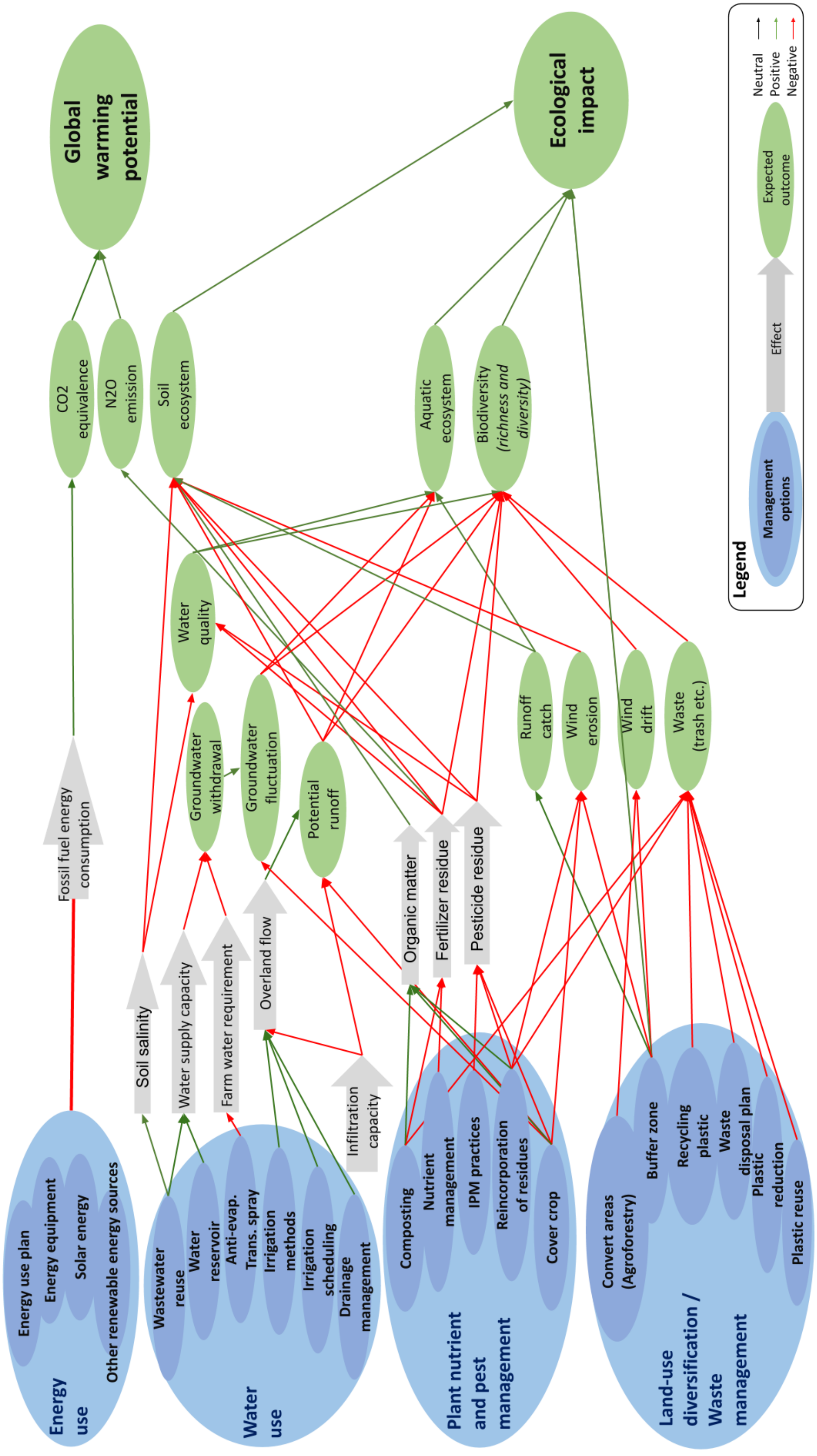

Figure S2: Detailed impact pathway representing the potential underlying variables modulating the impact on 21 certification measures on farm mitigation to climate change and farm ecology

Annex 3: Expert survey

The Action Alliance for Sustainable Bananas (ABNB) pledged to intensify its activities in the field of climate change adaptation and mitigation in the banana sector. Various measures to adapt to climate change effects are widely in place, some are already adopted by innovators and some are yet to be tested. ABNB wants to utilize the expertise and knowledge of experts in the field of climate change adaptation and mitigation in order to evaluate, which measures – that are currently available to most farmers – are most effective, cost-effective and pose the fewest risks.

Using Decision Analysis tools, the qualitative expertise and knowledge will be channeled and analyzed - quantifying individual knowledge and for making it measurable. The goal is to receive a prioritized list of measures, which should guide not only plantation owners and farmers but also certification schemes.

This questionnaire is to consult with banana and climate change experts to understand more about the banana production system as well as the potential of climate change adaptation and mitigation measures.

Please note that the questions refer to commercial banana production in humid regions. If you have any relevant resources or feedback, please kindly share in your answer or email cory.whitney@uni-bonn.de

1. My Name:………………………………………………

2. I would like to answer questions about:

   1. Buffer system, reforestation and system diversification *Skip to question 3*
   2. Irrigation and drainage *Skip to question 11*
   3. Pest management *Skip to question 31*
   4. Soil and plant nutrient *Skip to question 39*
   5. Waste management *Skip to question 45*
   6. Energy system *Skip to question 54*
   7. Extreme weather events and disasters *Skip to question 61*
   8. None of these *Skip to section 11 (Thank you for taking the time to fill in our survey!)*

I. Buffer system, reforestation and system diversification

3. In banana plantations, are buffer areas compulsory parts of the system? *Mark only one.*

   1. Yes 2. No 3. Other (specify) ____________

4. What do you think would be the best vegetation for buffer zones? *Choose all that apply.*

   1. Grasses 2. Shrubs 3. Timber trees

   4. Other (specify) ____________

5. How wide do you think the buffer areas around forests and waterways should be to be effective? Which factors most determine the width standards for buffer zone?

   ____

6. How can wind breaks be designed for banana plantations? *Choose all that apply.*

   1. Grow trees in buffer zone 2. Alley cropping 3. Trees planted scattered over the farm

   4. Other (specify) ____________

7. How do growers manage unproductive areas in their banana plantation? *Choose all that apply.*

   1. Leave it as natural habitat 2. Convert to other annual crops 3. Convert to agro-forestry

   4. Other (specify) ____________

8. Is it feasible for commercial banana growers to integrate shading trees into their production systems? *Mark only one.*

   1. Yes 2. No 3. Other (specify) ____________

9. Is crop diversification (e.g. intercropping or crop rotation) practical in large scale banana production? *Mark only one.*

   1. Yes 2. No 3. Other (specify) ____________

10. Please tell us about any additional benefits and obstacles for banana intercropping system?

    ____

II. Irrigation and drainage

11. What are the main sources of water that banana production relies on? *Choose all that apply.*

1. Rainfall 2. Ground water 3. Waste water

4. Surface water (rivers, lakes, ponds, streams) 5. Other (specify) ____________

12. To what extent is banana production reliant on rainfall? *Mark only one.*

	0	1	2	3	4	5	6	7	8	9	10	
Not at all	○	○	○	○	○	○	○	○	○	○	○	Totally dependent

13. To what extent is banana production reliant on groundwater for irrigation? *Mark only one.*

	0	1	2	3	4	5	6	7	8	9	10	
Not at all	○	○	○	○	○	○	○	○	○	○	○	Totally dependent

14. To what extent is banana production reliant on surface water (rivers, lakes, ponds, streams) for irrigation? *Mark only one.*

	0	1	2	3	4	5	6	7	8	9	10	
Not at all	○	○	○	○	○	○	○	○	○	○	○	Totally dependent

15. Is waste water used for irrigation in banana production? *Mark only one.*

	0	1	2	3	4	5	6	7	8	9	10	
Not at all	○	○	○	○	○	○	○	○	○	○	○	It is frequently used

16. Do banana production systems face water shortages? *Mark only one.*

1. Yes 2. No 3. Other (specify) ____________

17. What are common irrigation techniques used in large scale banana plantation? *Choose all that apply.*

1. Furrow irrigation 2. Flood irrigation 3. Drip irrigation

4. Under canopy single and series sprinkler irrigation 5. Overhead irrigation

6. Other (specify) ____________________

18. Rank the importance of the following irrigation techniques for banana plantations: *Choose all that apply.*

Rank	Furrow irrigation	Flood irrigation	Drip irrigation	Under canopy single and series sprinkler irrigation	Overhead irrigation
1st					
2nd					
3rd					
4th					
5th					
6th					
Not important					

19. How efficient are furrow irrigation techniques? *Mark only one..*

	0	1	2	3	4	5	6	7	8	9	10	
Not efficient at all	◯	◯	◯	◯	◯	◯	◯	◯	◯	◯	◯	Highly efficient

20. How efficient are flood irrigation techniques? *Mark only one.*

	0	1	2	3	4	5	6	7	8	9	10	
Not efficient at all	◯	◯	◯	◯	◯	◯	◯	◯	◯	◯	◯	Highly efficient

21. How efficient are drip irrigation techniques? *Mark only one.*

	0	1	2	3	4	5	6	7	8	9	10	
Not efficient at all	◯	◯	◯	◯	◯	◯	◯	◯	◯	◯	◯	Highly efficient

22. How efficient are "under canopy single and series sprinkler irrigation" techniques? *Mark only one.*

	0	1	2	3	4	5	6	7	8	9	10	
Not efficient at all	⬭	⬭	⬭	⬭	⬭	⬭	⬭	⬭	⬭	⬭	⬭	Highly efficient

23. How efficient are "overhead irrigation" techniques? *Mark only one.*

	0	1	2	3	4	5	6	7	8	9	10	
Not efficient at all	⬭	⬭	⬭	⬭	⬭	⬭	⬭	⬭	⬭	⬭	⬭	Highly efficient

24. How efficient are "other irrigation" techniques? *Mark only one.*

	0	1	2	3	4	5	6	7	8	9	10	
Not efficient at all	⬭	⬭	⬭	⬭	⬭	⬭	⬭	⬭	⬭	⬭	⬭	Highly efficient

25. Do you think drip irrigation is a feasible option for banana production system? *Mark only one.*

1. Yes 2. No 3. Other (specify) ____________________

26. Would antitranspirants effectively save water without compromising banana yield? *Mark only one.*

1. Yes 2. No 3. Other (specify) ____________________

27. What are the main risks of using wastewater from packing plant for irrigation? *Choose all that apply.*

1. Salinity 2. Toxicity 3. Other (specify) ____________________

28. What is a typical drainage system in banana plantation? *Mark only one*.

1. Open drainage 2. Under-ground drainage 3. Other (specify) ____________________

29. Are typical drainage system in banana plantation effective to avoid water- logging? *Mark only one.*

1. Yes 2. No 3. Other (specify) ____________________

30. How do you think drainage system should be designed to minimize possible risks (e.g. waterlog, flooding) for banana plantation? Please explain briefly

____

III. Pest management

31. Are Integrated Pest Management practices commonly used in pest control in commercial banana production? *Mark only one.*

1. Yes 2. No 3. Other (specify) ____________

32. How do farmers make spraying decision? *Choose all that apply.*

1. Based on regulations on spraying 2. Based on farm monitoring system
3. Mainly based on farmers' experiences 4. Other (specify) ____________

33. What are common practices for weeding? *Choose all that apply.*

1. Using chemical herbicide 2. Using organic herbicide 3. Soil cover
4. Manually 5. Mechanical equipment's 6. Other (specify) ____________

34. Which materials of ground cover do you think are practical and the most effective in the context of banana plantation? *Choose all that apply.*

1. Banana plant residues 2. Plastics or other synthesized materials
3. Cover crops 4. Other (specify) ____________

35. Can ground cover reduce weeds and the necessity for herbicides? *Mark only one.*

1. Yes 2. No 3. Other (specify) ____________

36. Do farmers remove suckers of banana plants? *Choose all that apply.*

1. Yes 2. No 3. Other (specify) ____________

37. How effective are mechanical and biological controls such as using mechanical traps and natural enemies for pest control comparing to chemical measure? *Mark only one*

1. More effective and labor intensive 2. Same effects and labor intensive
3. Less effective and labor intensive 4. Other (specify) ____________

38. Please list any important risks to using mechanical and biological methods.

____

IV. Soil and plant nutrients

39. How common is compost use in banana plantations? *Mark only one.*

	0	1	2	3	4	5	6	7	8	9	10	
None	◯	◯	◯	◯	◯	◯	◯	◯	◯	◯	◯	Very commonly applied

40. What are the common sources for composting materials in banana plantations? *Choose all that apply.*

1. Internal plant biomass from banana farm
2. External plant biomass from outside
3. Animal manures
4. Other (specify) ______

41. What is a more effective use of farm organic waste? *Choose all that apply.*

1. Composting 2. Returning to the field for soil cover 3. Other (specify) ______

42. How often is the soil tilled in banana plantations? *Choose all that apply.*

1. More than once a year
2. Once a year
3. Every two years
4. Once every 3-5 years
5. Other (specify) ______

43. Is reduced tillage necessary in current banana cultivation? *Choose all that apply.*

1. Yes 2. No 3. Other (specify) ______

44. What are the common methods for fertilizer application in banana production? *Choose all that apply.*

1. Through irrigation
2. Side dressing
3. Foliar application
4. Other (specify) ______

V. Waste management

45. What is the proportion of commercial banana growers having waste management stations?

____

46. At which production scale is a waste management station should be reasonable?

____

47. What could be the options to manage the plastic waste from banana plantation? *Choose all that apply*.

1. Reduction 2. Reutilization 3. Recycling 4. Landfill

5. Other (specify) ______________________________

VI. Energy system

48. Are there external renewable energy supply in the banana plantation area? *Mark only one.*

1. Yes 2. No 3. Other (specify) ____________

49. Where there is no external renewable energy supply, is it feasible to set up renewable energy systems on banana plantations? *Mark only one.*

1. Yes 2. No 3. Other (specify) ____________

50. Which types of renewable energy do you think feasible for a commercial banana farm? *Choose all that apply.*

1. Solar 2. Biomass 3. Wind 4. Hydropower 5. Geothermal

6. Other (specify) ____________

51. What are the main drawbacks to using solar energy for a banana farms? *Choose all that apply.*

1. High installation cost 2. Risk of power supply shortage 3. Weather dependent

4. Associated pollution 5. Other (specify) ____________

52. Is biomass energy a viable solution for banana plantations? *Mark only one.*

1. Yes 2. No 3. Other (specify) ____________

53. What are the main drawbacks to using biomass energy? *Choose all that apply.*

1. Availability of inputs 2. High cost 3. Resource trade-offs

4. Environment effects 5. Other (specify) ____________

54. What is the percentage of biomass energy, relative to total energy consumption, that would be reasonable to adopt? *Mark only one.*

	0	1	2	3	4	5	6	7	8	9	10	
0%	⬭	⬭	⬭	⬭	⬭	⬭	⬭	⬭	⬭	⬭	⬭	100%

II. Extreme weather events and disasters

55. Which weather events or disasters most likely to threaten banana production systems? *Choose all that apply.*

1. Cyclone/hurricane   2. Flooding   3. Drought Pest outbreak

4. Strong wind   5. Other (specify) ____________________

56. Which measures can mitigate the impacts of weather events and/or disasters that most likely to threaten banana production system?

____

57. Are there weather forecasting or early warning systems in place for banana production? *Mark only one.*

1. Yes   2. No   3. Other (specify) ____________________

58. Is it feasible to provide banana farmers with weather forecasts with agricultural advisories? *Mark only one.*

1. Yes   2. No   3. Other (specify) ____________________

59. Is weather indexed insurance applied by banana growers? If yes, is it a good option for reducing risks?

____

60. Are automated technical tools (e.g., GPS or remote sensing) used commonly in banana production? In which parts/activities these tools could be applicable?

____

Thank you for taking the time to fill in our survey!

References

Abdalla, M., A. Hastings, K. Cheng, Q. Yue, D. Chadwick, M. Espenberg, J. Truu, R. M. Rees, and P. Smith. 2019. "A Critical Review of the Impacts of Cover Crops on Nitrogen Leaching, Net Greenhouse Gas Balance and Crop Productivity." *Global Change Biology* 25 (8): 2530–43. https://doi.org/10.1111/gcb.14644.

Akcaoz, Handan. 2011. "Analysis of Energy Use for Banana Production: A Case Study from Turkey." *African Journal of Agricultural Research* 6 (25). https://doi.org/10.5897/AJAR09.480.

Aryal, D. R., V. Geissen, A. Ponce-Mendoza, R. R. Ramos-Reyes, and M. Becker. 2012. "Water Quality Under Intensive Banana Production and Extensive Pastureland in Tropical Mexico." *Journal of Plant Nutrition and Soil Science* 175: 553–59. https://doi.org/10.1002/jpln.201100117.

Barraza, Douglas, Kees Jansen, Berna van Wendel de Joode, and Catharina Wesseling. 2011. "Pesticide Use in Banana and Plantain Production and Risk Perception Among Local Actors in Talamanca, Costa Rica." *Environmental Research* 111 (5): 708–17. https://doi.org/https://doi.org/10.1016/j.envres.2011.02.009.

Barraza, Douglas, Kees Jansen, Catharina Wesseling, and Berna van Wendel de Joode. 2020. "Pesticide Risk Perceptions Among Bystanders of Aerial Spraying on Bananas in Costa Rica." *Environmental Research* 189: 109877. https://doi.org/https://doi.org/10.1016/j.envres.2020.109877.

Bekunda, M. A., and P. L. Woomer. 1996. "Organic Resource Management in Banana-Based Cropping Systems of the Lake Victoria Basin, Uganda." *Agriculture, Ecosystems & Environment* 59 (3): 171–80. https://doi.org/10.1016/0167-8809(96)01057-2.

Bellamy, A. S. 2013. "Banana Production Systems: Identification of Alternative Systems for More Sustainable Production." *AMBIO* 42: 334–43. https://doi.org/10.1007/s13280-012-0341-y.

Blazy, Jean-Marc, Marc Dorel, Frédéric Salmon, Harry Ozier-Lafontaine, Jacques Wery, and Philippe Tixier. 2009. "Model-Based Assessment of Technological Innovation in Banana Cropping Systems Contextualized by Farm Types in Guadeloupe." *European Journal of Agronomy* 31 (1): 10–19. https://doi.org/https://doi.org/10.1016/j.eja.2009.02.001.

Castillo, Luisa Eugenia, Eduardo Martínez, Clemens Ruepert, Candida Savage, Michael Gilek, Margareth Pinnock, and Efrain Solis. 2006. "Water Quality and Macroinvertebrate Community Response Following Pesticide Applications in a Banana Plantation, Limon, Costa Rica." *Science of The Total Environment* 367 (1): 418–32. https://doi.org/https://doi.org/10.1016/j.scitotenv.2006.02.052.

Chaves, Alicia, Damian Shea, and W. Gregory Cope. 2007. "Environmental Fate of Chlorothalonil in a Costa Rican Banana Plantation." *Chemosphere* 69 (7): 1166–74. https://doi.org/https://doi.org/10.1016/j.chemosphere.2007.03.048.

Collentine, Dennis, Holger Johnsson, Peter Larsson, Hampus Markensten, and Kristian Persson. 2015. "Designing Cost Efficient Buffer Zone Programs: An Application of the FyrisSKZ Tool in a Swedish Catchment." *AMBIO* 44 (S2): 311–18. https://doi.org/10.1007/s13280-015-0627-y.

Coltro, Leda, and Thiago U. Karaski. 2019. "Environmental Indicators of Banana Production in Brazil: Cavendish and Prata Varieties." *Journal of Cleaner Production* 207 (January): 363–78. https://doi.org/10.1016/j.jclepro.2018.09.258.

Côte, F. X., C. Abadie, R. Achard, P. Cattan, C. Chabrier, M. Dorel, L. de Lapeyre de Bellaire, J. M. Risède, F. Salmon, and P. Tixier. 2009. "Integrated Pest Management Approaches Developed in the French West Indies to Reduce Pesticide Use in Banana Production Systems." *Acta Horticulturae*, no. 828 (May): 375–82. https://doi.org/10.17660/ActaHortic.2009.828.38.

de Oliveira, Aureo S, Ricardo Trezza, Eduardo Holzapfel, Ignacio Lorite, and Vital Pedro S Paz. 2009. "Irrigation Water Management in Latin America." *Chilean Journal of Agricultural Research* 69 (December). https://doi.org/10.4067/S0718-58392009000500002.

Ducros, C., and C. Joyce. 2003. "Field-Based Evaluation Tool for Riparian Buffer Zones in Agricultural Catchments." *Environmental Management* 32 (2): 252–67. https://doi.org/10.1007/s00267-003-2913-x.

El-Kader, A. 2006. "Effect of Soil Moisture Levels and Some Antitranspirants on Vegetative Growth, Leaf Mineral Content, Yield and Fruit Quality of Williams Banana Plants," 9.

Gawad, Nehad. 2014. "Effect of Some Anti-Transpirant to Reduce Amount of Irrigation Water Added to the Banana Cv."grand Nain" in Sandy Soil." *Egyptian Journal of Horticulture* 42 (Issue 1): 69–86. https://doi.org/10.21608/ejoh.2014.1181.

Henriques, W., R. D. Jeffers, T. E. Lacher, and R. J. Kendall. 1997. "Agrochemical Use on Banana Plantations in Latin America: Perspectives on Ecological Risk." *Environmental Toxicology and Chemistry* 16 (1): 91–99. https://doi.org/10.1002/etc.5620160110.

Iriarte, Alfredo. 2014. "Carbon Footprint of Premium Quality Export Bananas: Case Study in Ecuador, the World's Largest Exporter." *Science of the Total Environment*, 7.

Israeli, Y., J. Hagin, and S. Katz. 1985. "Efficiency of Fertilizers as Nitrogen Sources to Banana Plantations Under Drip Irrigation." *Fertilizer Research* 8 (2): 101–6. https://doi.org/10.1007/BF01048893.

Jacobs, David, Natacha Marzolf, Juan Roberto Paredes, Wilson Rickerson, Hilary Flynn, Christina Becker-Birck, and Mauricio Solano-Peralta. 2013. "Analysis of Renewable Energy Incentives in the Latin America and Caribbean Region: The Feed-in Tariff Case." *Energy Policy* 60 (September): 601–10. https://doi.org/10.1016/j.enpol.2012.09.024.

Johns, Gg. 1994. "Effect of Arachis Pintoi Groundcover on Performance of Bananas in Northern New South Wales." *Australian Journal of Experimental Agriculture* 34 (8): 1197. https://doi.org/10.1071/EA9941197.

Keshavan, G., M. Kavino, and V. Ponnuswami. 2011. "Influence of Different Nitrogen Sources and Levels on Yield and Quality of Banana (*Musa Spp* .)." *Archives of Agronomy and Soil Science* 57 (3): 305–15. https://doi.org/10.1080/03650340903302286.

Kukulies, T., A. Pattison, L. Forsyth, and P. Nelson. 2014. "Integrating Organic Matter into Banana Plantation in North Queensland: The Effects on Soil Properties." *Acta Horticulturae*, no. 1018 (January): 441–47. https://doi.org/10.17660/ActaHortic.2014.1018.48.

Lavigne, C., R. Achard, P. Tixier, and M. Lesueur Jannoyer. 2012. "How to Integrate Cover Crops to Enhance Sustainability in Banana and Citrus Cropping Systems." *Acta Horticulturae*, no. 928 (February): 351–57. https://doi.org/10.17660/ActaHortic.2012.928.47.

Lin, Te-Sheng, Chun-Nan Lin, and Ke-Chung Peng. 2019. "Comparative Analysis of Production Costs and Revenue on the Banana Planting Season in Taiwan." *OALib* 06 (01): 1–8. https://doi.org/10.4236/oalib.1105180.

Lobell, D. B. 2007. "The Cost of Uncertainty for Nitrogen Fertilizer Management: A Sensitivity Analysis." *Field Crops Research* 100 (2-3): 210–17. https://doi.org/10.1016/j.fcr.2006.07.007.

Luedeling, Eike, Lutz Goehring, Katja Schiffers, Cory Whitney, and Eduardo Fernandez. 2022. *decisionSupport: Quantitative Support of Decision Making Under Uncertainty.* http://www.worldagroforestry.org/.

Masters, B. L. 2019. "Nitrous Oxide Emissions from Soil in Mango and Banana Fields: Effects of Nitrogen Rate, Fertiliser Type, and Ground Cover Practices." https://doi.org/10.25903/5E545C1348B4B.

McKergow, L., I. Prosser, R. Grayson, D. Weaver, and D. Heiner. 2004. "Grass or Trees? Performance of Riparian Buffers Under Natural Rainfall Conditions, Australia." *American Water Resources Association*, 7.

McVittie, Alistair, Lisa Norton, Julia Martin-Ortega, Ioanna Siameti, Klaus Glenk, and Inge Aalders. 2015. "Operationalizing an Ecosystem Services-Based Approach Using Bayesian Belief Networks: An Application to Riparian Buffer Strips." *Ecological Economics* 110 (February): 15–27. https://doi.org/10.1016/j.ecolecon.2014.12.004.

Melo, Cristian J., and Steven A. Wolf. 2005. "Empirical Assessment of Eco-Certification: The Case of Ecuadorian Bananas." *Organization & Environment* 18 (3): 287–317. https://doi.org/10.1177/1086026605279461.

Meya, Akida I., Patrick A. Ndakidemi, Kelvin M. Mtei, Rony Swennen, and Roel Merckx. 2020. "Optimizing Soil Fertility Management Strategies to Enhance Banana Production in Volcanic Soils of the Northern Highlands, Tanzania." *Agronomy* 10 (2): 289. https://doi.org/10.3390/agronomy10020289.

Minhas, P. S., Tiago B. Ramos, Alon Ben-Gal, and Luis S. Pereira. 2020. "Coping with Salinity in Irrigated Agriculture: Crop Evapotranspiration and Water Management Issues." *Agricultural Water Management* 227 (January): 105832. https://doi.org/10.1016/j.agwat.2019.105832.

Mugerwa, Nathan. 2007. "Rainwater Harvesting and Rural Livelihood Improvement in Banana Growing Areas of Uganda." *Department of Water and Environmental Studies, Linköping University*, no. Master thesis.

Muscutt, A. D., G. L. Harris, S. W. Bailey, and D. B. Davies. 1993. "Buffer Zones to Improve Water Quality: A Review of Their Potential Use in UK Agriculture." *Agriculture, Ecosystems & Environment* 45 (1-2): 59–77. https://doi.org/10.1016/0167-8809(93)90059-X.

Ouédraogo, E. 2001. "Use of Compost to Improve Soil Properties and Crop Productivity Under Low Input Agricultural System in West Africa." *Agriculture, Ecosystems & Environment* 84 (3): 259–66. https://doi.org/10.1016/S0167-8809(00)00246-2.

Panigrahi, N., A. J. Thompson, S. Zubelzu, and J. W. Knox. 2021. "Identifying Opportunities to Improve Management of Water Stress in Banana Production." *Scientia Horticulturae* 276 (January): 109735. https://doi.org/10.1016/j.scienta.2020.109735.

Panigrahi, P., S. Raychaudhuri, A. K. Thakur, A. K. Nayak, P. Sahu, and S. K. Ambast. 2019. "Automatic Drip Irrigation Scheduling Effects on Yield and Water Productivity of Banana." *Scientia Horticulturae* 257 (November): 108677. https://doi.org/10.1016/j.scienta.2019.108677.

Păunescu, Carmen, and Laura Blid. 2016. "Effective Energy Planning for Improving the Enterprise's Energy Performance." *Management & Marketing* 11 (3): 512–31. https://doi.org/10.1515/mmcks-2016-0013.

Pawar, D. D., S. K. Dingre, and P. G. Bhoi. 2017. "Productivity and Economics of Drip Irrigated Banana (Musa

Spp.) Under Different Planting and Fertigation Techniques in Sub Tropical India." *Communications in Soil Science and Plant Analysis*, February, 00103624.2017.1282505. https://doi.org/10.1080/00103624.2017.1282505.

Pogson, Mark, Astley Hastings, and Pete Smith. 2013. "How Does Bioenergy Compare with Other Land-Based Renewable Energy Sources Globally?" *GCB Bioenergy* 5 (5): 513–24. https://doi.org/10.1111/gcbb.12013.

Pramanik, Sanjit, and Sanmay Kumar Patra. 2016. "Growth, Yield, Quality and Irrigation Water Use Efficiency of Banana Under Drip Irrigation and Fertigation in the Gangetic Plain of West Bengal," 10.

Quaresma, Mateus Augusto Lima, Fábio Luiz De Oliveira, and Diego Mathias Natal Da Silva. 2017. "Leguminous Cover Crops for Banana Plantations in Semi-Arid Regions." *Revista Caatinga* 30 (3): 614–21. https://doi.org/10.1590/1983-21252017v30n309rc.

Rahman, H. M. T., J. C. Deb, G. M. Hickey, and I. Kayes. 2014. "Contrasting the Financial Efficiency of Agroforestry Practices in Buffer Zone Management of Madhupur National Park, Bangladesh." *Journal of Forest Research* 19 (1): 12–21. https://doi.org/10.1007/s10310-013-0392-3.

Rowlings, D. W., P. R. Grace, C. Scheer, and R. Kiese. 2013. "Influence of Nitrogen Fertiliser Application and Timing on Greenhouse Gas Emissions from a Lychee (*Litchi Chinensis*) Orchard in Humid Subtropical Australia." *Agriculture, Ecosystems & Environment* 179 (October): 168–78. https://doi.org/10.1016/j.agee.2013.08.013.

Russo, R. O., and C. Hernández. 1995. "The Environmental Impact of Banana Production Can Be Diminished by Proper Treatment of Wastes." *Journal of Sustainable Agriculture* 5 (3): 5–13. https://doi.org/10.1300/J064v05n03_03.

Sarath, S., K. Uma, and P. Naveen Kumar. 2017. "A Study on Energy Use Pattern for Banana Production in Erode District of Tamil Nadu." *Madras Agricultural Journal* 104 (10-12): 372. https://doi.org/10.29321/MAJ.2017.000081.

Stover, R. H. 1986. "Disease Management Strategies and the Survival of the Banana Industry." *Ann. Rev. Phytopathol*, 9.

Strobl, Eric, and Preeya Mohan. 2020. "Climate and the Global Spread and Impact of Bananas' Black Leaf Sigatoka Disease." *Atmosphere* 11 (9): 947. https://doi.org/10.3390/atmos11090947.

Svanes, Erik, and Anna K. S. Aronsson. 2013. "Carbon Footprint of a Cavendish Banana Supply Chain." *The International Journal of Life Cycle Assessment* 18 (8): 1450–64. https://doi.org/10.1007/s11367-013-0602-4.

Svensson, O., A. S. Bellamy, P. J. Van den Brink, M. Tedengren, and J. S. Gunnarsson. 2018. "Assessing the Ecological Impact of Banana Farms on Water Quality Using Aquatic Macroinvertebrate Community Composition." *Environmental Science and Pollution Research* 25 (14): 13373–81. https://doi.org/10.1007/s11356-016-8248-y.

Tock, Jing Yan, Chin Lin Lai, Keat Teong Lee, Kok Tat Tan, and Subhash Bhatia. 2010. "Banana Biomass as Potential Renewable Energy Resource: A Malaysian Case Study." *Renewable and Sustainable Energy Reviews* 14 (2): 798–805. https://doi.org/10.1016/j.rser.2009.10.010.

Tursun, Nihat, Doğan Işık, Zeynep Demir, and Khawar Jabran. 2018. "Use of Living, Mowed, and Soil-Incorporated Cover Crops for Weed Control in Apricot Orchards." *Agronomy* 8 (8): 150. https://doi.org/10.3390/agronomy8080150.

Vallejo-Chaverri, A. L., M. A. Vallejo-Solís, J. Nájera-Fernández, and L. A. Garnier-Zamora. 2018. "Methodological Guide to Reduce Carbon and Water Footprints in Banana Plantations." *Acción Clima II Project – FAO and GIZ* 140.

van Asten, P. J. A., A. M. Fermont, and G. Taulya. 2011. "Drought Is a Major Yield Loss Factor for Rainfed East African Highland Banana." *Agricultural Water Management* 98 (4): 541–52. https://doi.org/10.1016/j.agwat.2010.10.005.

Veldkamp, E., and M. Keller. 1997. "Nitrogen Oxide Emissions from a Banana Plantation in the Humid Tropics." *Journal of Geophysical Research: Atmospheres* 102 (D13): 15889–98. https://doi.org/10.1029/97JD00767.

Viviescas, Cindy, Lucas Lima, Fabio A. Diuana, Eveline Vasquez, Camila Ludovique, Gabriela N. Silva, Vanessa Huback, et al. 2019. "Contribution of Variable Renewable Energy to Increase Energy Security in Latin America: Complementarity and Climate Change Impacts on Wind and Solar Resources." *Renewable and Sustainable Energy Reviews* 113 (October): 109232. https://doi.org/10.1016/j.rser.2019.06.039.

Wairegi, L. W. I., and P. J. A. van Asten. 2010. "The Agronomic and Economic Benefits of Fertilizer and Mulch Use in Highland Banana Systems in Uganda." *Agricultural Systems* 103 (8): 543–50. https://doi.org/10.1016/j.agsy.2010.06.002.

Zhu, Yueji, Qi Yang, and Cheng Zhang. 2021. "Adaptation Strategies and Land Productivity of Banana Farmers Under Climate Change in China." *Climate Risk Management* 34: 100368. https://doi.org/10.1016/j.crm.2021.100368.
